# Epigenetic state determines the in vivo efficacy of STING agonist therapy

**DOI:** 10.1038/s41467-023-37217-1

**Published:** 2023-03-22

**Authors:** Rana Falahat, Anders Berglund, Patricio Perez-Villarroel, Ryan M. Putney, Imene Hamaidi, Sungjune Kim, Shari Pilon-Thomas, Glen N. Barber, James J. Mulé

**Affiliations:** 1 Department of Immunology, Moffitt Cancer Center, Tampa, FL 33612 USA; 2 Department of Biostatistics and Bioinformatics, Moffitt Cancer Center, Tampa, FL 33612 USA; 3 Radiation Oncology Program, Moffitt Cancer Center, Tampa, FL 33612 USA; 4 Cutaneous Oncology Program, Moffitt Cancer Center, Tampa, FL 33612 USA; 5 Department of Cell Biology, University of Miami Miller School of Medicine, Miami, FL 33136 USA

**Keywords:** Tumour immunology, Melanoma

## Abstract

While STING-activating agents have shown limited efficacy in early-phase clinical trials, multiple lines of evidence suggest the importance of tumor cell-intrinsic STING function in mediating antitumor immune responses. Although STING signaling is impaired in human melanoma, its restoration through epigenetic reprogramming can augment its antigenicity and T cell recognition. In this study, we show that reversal of methylation silencing of STING in murine melanoma cell lines using a clinically available DNA methylation inhibitor can improve agonist-induced STING activation and type-I IFN induction, which, in tumor-bearing mice, can induce tumor regression through a CD8^+^ T cell-dependent immune response. These findings not only provide mechanistic insight into how STING signaling dysfunction in tumor cells can contribute to impaired responses to STING agonist therapy, but also suggest that pharmacological restoration of STING signaling through epigenetic reprogramming might improve the therapeutic efficacy of STING agonists.

## Abstract (web summary)

STING agonists have shown limited efficacy in early-phase clinical trials despite promising pre-clinical data. This study shows the potential clinical relevance of the use of combination STING agonists and demethylating agent therapies to induce the expression of STING.

## Introduction

Although cancer immunotherapies, including adoptive cell transfer of tumor-infiltrating lymphocytes and immune checkpoint inhibitor antibodies can mediate durable tumor regression in patients with metastatic melanoma, their efficacy remains limited. Therefore, a thorough understanding of the tumor immune escape mechanisms is needed to overcome resistance to cancer immunotherapies. Tumors cells can escape the host’s immune recognition through a variety of mechanisms involving both tumor cell-intrinsic and -extrinsic elements^1–3^. Although targeting defective T cell function either by blocking of the immune inhibitory molecules or through reconstitution of tumor-reactive T cells using adoptive cell transfer has been the focus of current immunotherapies^4,5^, therapeutic resistance, in certain patients both primary and acquired, stresses the need to identify and target other contributing elements^6–9^.

Lack of tumor antigenicity and insufficient recruitment and infiltration of T cells into tumors are, in part, two hallmarks in patients who do not respond favorably to immunotherapies^10–13^. While a clear understanding of molecular mechanisms governing immunotherapy responsiveness is still evolving, multiple clinical observations have linked intratumoral presence of CD8^+^ T cells to a type-I interferon (IFN) transcriptional signature^14–16^. Similarly, several preclinical studies have confirmed the important function of type-I IFNs in the initiation of spontaneous and iatrogenic antitumor immune T cell responses by highlighting their role in triggering a sequence of autocrine and paracrine signaling events that lead to enhanced expression of MHC molecules and induction of T cell homing chemokines within the tumors^16,17^. Among different upstream pathways that drive type-I IFN induction, the stimulator of interferon genes (STING) has been shown to be a major pathway for the detection of immunogenic tumors and initiation of a spontaneous T cell response^18–21^. This finding has inspired studies to evaluate whether direct activation of this pathway using pharmacologic STING agonists could be used in facilitating antitumor immune responses^22–25^. While clear therapeutic activity of STING agonists has been shown in preclinical tumor models, early-phase clinical trials with these agents in cancer patients, either as monotherapy or in combination with immune checkpoint inhibitors, have so far shown lack of efficacy^26–28^. Therefore, uncovering mechanistic details of STING pathway activation could potentially improve antitumor immunity.

Another aspect of the activation of STING signaling in melanoma cells is the downstream induction of CXCL10 and CCL5^29^. These chemokines have been identified as the dominant mediators for the recruitment of CXCR3^+^ and CCR5^+^ tumor-specific T lymphocytes into the tumors and their intratumoral expression correlated with favorable clinical outcomes in patients with melanoma^30–32^. Notably, they are also included in a 12-chemokine gene expression signature that we have shown to uncover the presence of the tumor-localized, tertiary lymphoid structures, which are found to positively correlate with overall survival in certain patients with metastatic melanoma^33^. However, because it is unclear how STING-mediated induction of these chemokines by tumor cells per se modulates the T cell microenvironment towards a positive prognostic outcome, it is of biological and clinical relevance to ascertain the underlying, operative mechanism(s).

Although antigen-presenting cells (APCs) participate in STING-dependent antitumor immune responses^18,22^, recent studies have provided compelling evidence that tumor cell-intrinsic STING activity is also an important contributor^34–36^. Indeed, we previously reported that activation of STING signaling in human melanoma cell lines can increase their antigenicity by upregulating MHC class I molecules, leading to more effective immune recognition and antigen presentation to tumor-infiltrating lymphocytes (TIL)^29^. However, we have also shown that there is a widespread impairment of STING signaling in human melanoma cell lines, which can limit their recognition, and sensitivity to, TIL-mediated killing. In addition, we have also demonstrated using genome-wide DNA methylation profiling that promoter hypermethylation of cGAS and STING leads to coordinated transcriptional silencing of these genes, thereby suppressing STING signaling in human melanoma and melanoma cell lines^37^. Thus, it may be hypothesized that defective STING signaling in tumor cells could represent a mechanism of escape from immune recognition and elimination, as well as resistance to STING agonist- and TIL-based immunotherapies.

Here, we use mouse models of melanoma with defective STING signaling, either in the host immune component or in the tumor itself, to show that a DNA methyltransferase inhibitor can rescue tumor cell-intrinsic STING signaling dysfunction in vivo and subsequently induce greater tumor regression through a CD8^+^ T cell-dependent mechanism. These findings establish a critical role in vivo for an active STING pathway in tumor cells in shaping the therapeutic response to STING agonists.

## Results

### Melanoma cell-intrinsic STING activity alone is insufficient for durable tumor control

**Fig. 1:**
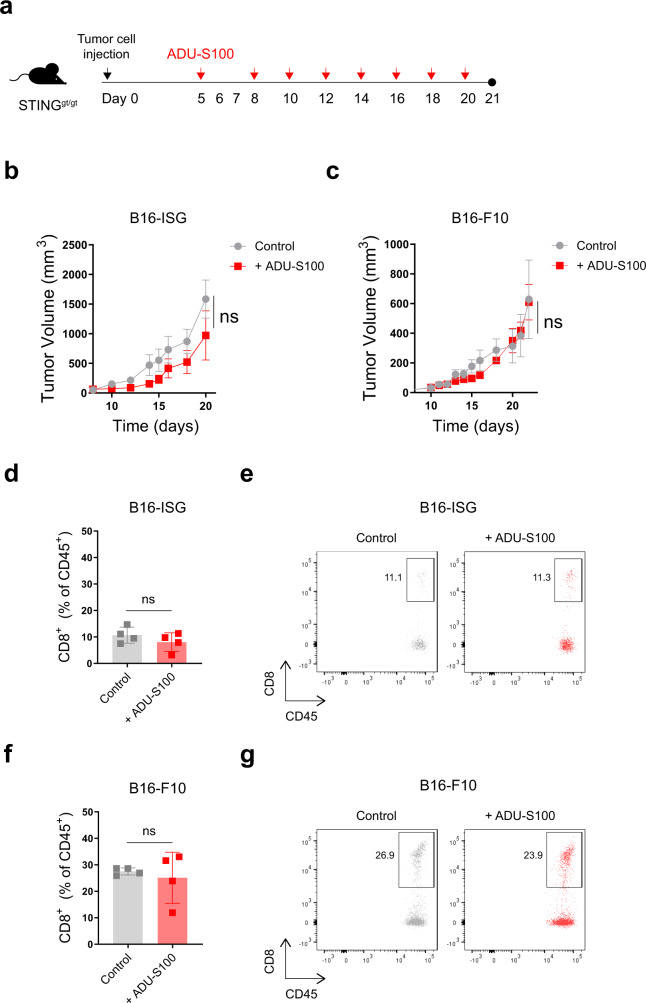
Melanoma cell-intrinsic STING activity alone is insufficient for durable tumor control. Schematic of the STING agonist treatment schedule. Groups of STING^gt/gt^ mice were injected subcutaneously with 1.5 × 10^5^ B16-ISG or B16-F10 on day 0. On days 5, 8, 10, 12, 14, 16, 18 and 20 following tumor injection, tumor-bearing mice were intratumorally treated with either PBS or 50 μg ADU-S100 (**a**). Tumor growth curves of B16-ISG (**b**) and B16-F10 (**c**) in STING^gt/gt^ mice treated with PBS control or ADU-S100 as indicated in (**a**). Data are shown as the mean ± SEM and are representative of two independent experiments. *n* = 4 and 5 mice in (**b**) and *n* = 5 and 6 mice in (**c**) for ADU-S100 and Control groups, respectively. The frequency of CD8^+^ cells within the CD45^+^ population in B16-ISG (**d**) and B16-F10 (**f**) tumors treated with PBS control or ADU-S100 (*n* = 4 mice per group). Data are shown as mean ± SD and are representative of two independent experiments. Statistical significance was determined by a two-sided t test (ns, not significant). Representative flow cytometry plots for **d** and **f** are shown in **e** and **g**, respectively.

To investigate whether tumor cell-intrinsic STING activity can influence antitumor responses to STING agonist therapy, we injected STING^gt/gt^ mice subcutaneously with B16-ISG or B16-ISG-STING^KO^ murine melanoma cells and treated them intratumorally with either the STING agonist ADU-S100 or PBS as vehicle control (Supplementary Fig. 1a). This approach allowed us to separate the effect of tumor cell-intrinsic STING activity from that driven by the host immune system. Within the second week of tumor injection, while we did not find a significant difference in the tumor growth between the PBS-treated B16-ISG and B16-ISG-STING^KO^ tumors, ADU-S100 treatment of the B16-ISG tumors delayed their growth in STING^gt/gt^ mice (Supplementary Fig. 1b). In contrast, ADU-S100 treatment did not impact B16-ISG-STING^KO^ tumor growth. Furthermore, flow cytometry analysis of the TIL on day 14 indicated a significant increase in the frequency and a total number of CD8^+^ T cells in STING agonist-treated B16-ISG tumors compared to their control group (Supplementary Fig. 1c, d) but not in B16-ISG-STING^KO^ tumors. Similar to B16-ISG, STING agonist treatment also delayed tumor growth in two different mouse models of melanoma, B16-F10, and Yumm1.7, on day 14 (Supplementary Fig. 1e). These results show that melanoma-intrinsic STING activity can contribute to T cell priming and T cell tumor trafficking in the low tumor burden state. However, when we performed an analogous experiment with a longer duration, by day 20, ADU-S100 lost the ability to maintain tumor control in B16-ISG tumors (Fig. 1a, b). Similar results were obtained with a B16-F10 model in STING^gt/gt^ mice (Fig. 1c). Moreover, we did not find a significant difference in the frequency of CD8^+^ T cells infiltrating either B16-ISG or B16-F10 ADU-S100-treated tumors compared to their controls (Fig. 1d–g). Collectively, these results indicate that although melanoma-intrinsic STING activity can mediate antitumor immune responses at the early stages of tumor growth (1–2 weeks), it is insufficient to mediate long-lasting tumor control in response to treatment with a STING agonist.

### Promoter hypermethylation suppresses STING expression in mouse melanoma cell lines

**Fig. 2:**
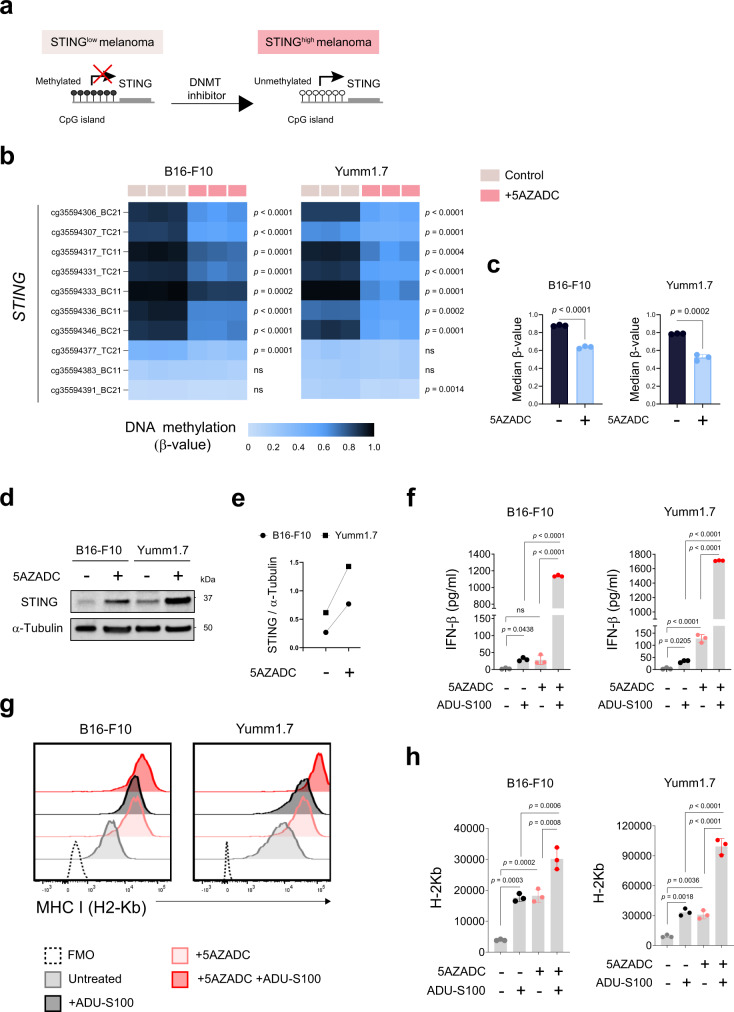
Promoter hypermethylation suppresses STING expression in mouse melanoma cell lines. Schematic of hypermethylation-mediated STING silencing and its reversal through epigenetic reprogramming in STING^low^ melanoma cells (**a**). β-value heat map showing DNA methylation levels across ten *STING* CpG probes in B16-F10 and Yumm1.7 mouse melanoma cell lines with and without 5AZADC treatment (**b**). Median β-values of ten *STING* CpG probes in B16-F10 and Yumm1.7 cells ± 5AZADC. Data are shown as mean ± SD (*n* = 3 biological replicates) and are representative of two independent experiments (**c**). Immunoblot analysis of STING expression in B16-F10 and Yumm1.7 cells with or without 5AZADC treatment. α-tubulin was used as a loading control (**d**). Images are representative of three independent experiments. The ratio of total STING to α-tubulin for each cell line with or without 5AZADC treatment (**e**). Following 5AZADC treatment, B16-F10, and Yumm1.7 cells were stimulated with the STING agonist ADU-S100 for 24 h. Induction of IFN-β in cell culture supernatants was measured using ELISA and reported as mean ± SD (*n* = 3 biological replicates) (**f**). Representative histograms (**g**) and mean fluorescence intensity (MFI) of MHC I (H2-Kb) expression on indicated cell lines (*n* = 3 biological replicates). Data are shown as the mean ± SD (**h**). Data are representative of three independent experiments (**f**–**h**). Statistical significance was determined by a two-sided t-test (**b** and **c**) and one-way ANOVA (**f**and **h**) (ns, not significant).

We have recently reported that STING expression is downregulated or lost in a notable subset of human melanoma cell lines mainly through promoter hypermethylation-driven silencing^29,37^, which can be restored through pharmacologic inhibition of DNA methylation (Fig. 2a). These findings led us to hypothesize that hypermethylation of the STING promoter also in mouse melanoma cell lines can mediate their coordinated transcriptional silencing and contribute to the impairment of the STING signaling function. To test this, we performed genome-wide DNA methylation profiling using the Infinium Mouse Methylation BeadChip microarray in B16-F10 and Yumm1.7 melanoma cell lines before and after their treatment with DNA methyltransferase inhibitor 5-aza-2’-deoxycytidine (5AZADC, decitabine). While we observed hypermethylation of STING promoter as indicated by β-values in both cell lines, their treatment with 5AZADC resulted in a decrease in STING promoter methylation levels (Fig. 2b, c). We also found reconstitution of STING protein expression in both B16-F10 and Yumm1.7 cell lines following their treatment with 5AZADC (Fig. 2d, e) further suggesting promoter hypermethylation-driven STING silencing and indicating their similarities in epigenetic regulation of STING to human melanoma cell lines. To further evaluate whether demethylation could restore functional activation of STING signaling, following 5AZADC treatment we next stimulated these cell lines with ADU-S100 and assessed STING-dependent downstream effects. We observed up to a 46-fold increase in induction of IFN-β (*p* < 0.0001) (Fig. 2f) and a notable increase in MHC class I surface expression in 5AZADC-pretreated B16-F10 and Yumm1.7 cells (Fig. 2g, h) compared to untreated controls following their exposure to ADU-S100. Although to a lesser extent, we also observed 5AZADC-mediated induction of IFN-β and upregulation of MHC class I in both B16-F10 and Yumm1.7 cell lines (Fig. 2f–h).

Consistent with our findings of 5AZADC-triggered IFN-β expression, multiple studies have shown that DNA demethylating agents among other epigenetic modulators can induce a type I IFN response in cancer cells through the de-repression of endogenous retroviruses (ERVs) and activation of the MDA5-MAVS pathway^38–40^. To determine whether the upregulation of MHC class I in response to treatment with 5AZADC occurs through type I IFN signaling, we blocked type I IFN receptor using an IFNAR blocking antibody in B16-F10 and Yumm1.7 cells. Indeed, in both cell lines blockade of IFNAR inhibited 5AZADC-induced upregulation of MHC class I (Supplementary Fig. 2a, b) as well as upregulation of LMP2 and TAP1, two key components of MHC class I antigen-processing pathway (Supplementary Fig. 2c, d).

To evaluate whether the profound effect of 5AZADC-pretreatment and ADU-S100 stimulation on IFN-β induction and MHC class I upregulation was driven by activation of STING signaling, we performed analogous experiments using B16-ISG and B16-ISG-STING^KO^ cells (Supplementary Fig. 3a). Similar to our observations in B16-F10 and Yumm1.7 cells, we found a 36-fold increase in induction of IFN-β (*p* < 0.0001) (Supplementary Fig. 3b) as well as a 4.4-fold increase in surface expression of MHC class I (*p* < 0.0001) (Supplementary Fig. 3c, d) in 5AZADC-pretreated B16-ISG cells in response to stimulation with the STING agonist, but not in 5AZADC-pretreated B16-ISG-STING^KO^ cells. Although we observed 5AZADC-induced upregulation of MHC class I in both B16-ISG and B16-ISG-STING^KO^ cell lines (Supplementary Fig. 3c, d), unlike 5AZADC-pretreated B16-ISG cells, further stimulation with the STING agonist did not increase surface expression of MHC class I in 5AZADC-pretreated B16-ISG-STING^KO^ cells, suggesting that DNA demethylation-induced increased IFN-β production and upregulation of MHC I in response to agonist stimulation in melanoma cells is STING mediated. Collectively, these data indicate that demethylation could enhance functional activation of STING signaling in mouse melanoma cell lines.

### DNMT3A and DNMT3B are involved in melanoma STING silencing

**Fig. 3:**
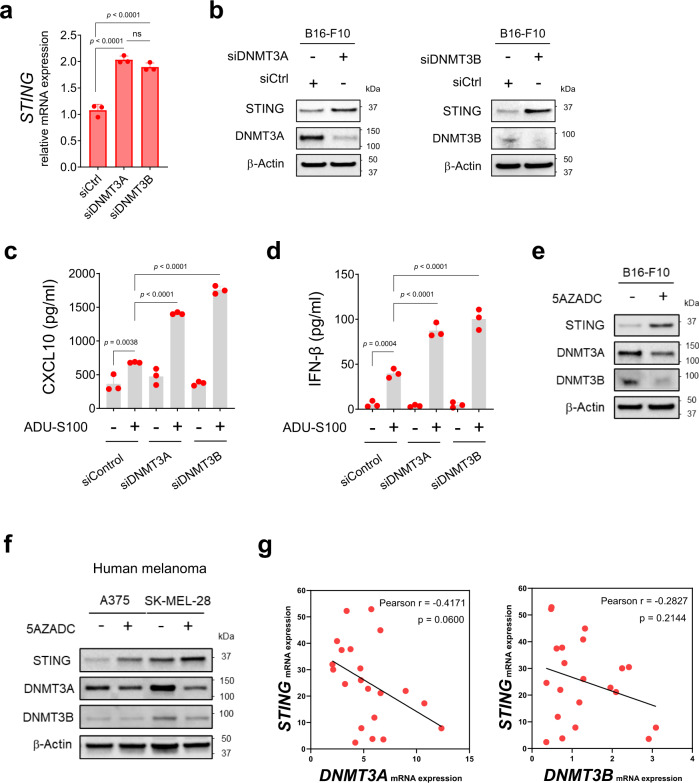
DNMT3A and DNMT3B are involved in STING silencing in melanoma. Quantitative RT-PCR analysis of *STING* mRNA expression in transfected B16-F10 cells with siRNA specific for DNMT3A (siDNMT3A) or DNMT3B (siDNMT3B) or nontarget siRNA (siControl) (*n* = 3). Data are shown as mean ± SD and are representative of two independent experiments. Statistical significance was determined using one-way ANOVA (**a**) (ns, not significant). Immunoblot analysis of STING, DNMT3A, and DNMT3B expression in indicated cells. β-Actin was used as a loading control (**b**). Levels of CXCL10 (**c**) and IFN-β (**d**) in cell culture supernatants measured using ELISA and reported as mean ± SD (*n* = 3 biological replicates). Data are representative of two independent experiments (**c**, **d**). Statistical significance was determined by one-way ANOVA. Immunoblot analysis of STING, DNMT3A and DNMT3B expression in B16-F10 (**e**) and A375 and SK-MEL-28 human melanoma cell lines (**f**) with or without 5AZADC treatment. Images in (**b**) and (**e**–**f**) are representative of three independent experiments. Correlative analysis of *STING* mRNA expression with *DNMT3A* and *DNMT3B* in metastatic melanoma samples (*n* = 21) from cBioPortal database using Pearson’s correlation coefficient. P-values shown are two-sided P-values derived from the Pearson correlation test (**g**).

The observation of promoter hypermethylation-mediated STING suppression in human^37^ and mouse melanoma cell lines (Fig. 2b, c) suggested a possible epigenetic mechanism driven by DNA methyltransferases (DNMTs) including DNMT1, DNMT3A, and DNMT3B. To further interrogate the role of DNMTs in melanoma-STING silencing, we transfected B16-F10 cells with small interfering RNA (siRNA) targeting DNMT1, DNMT3A, and DNMT3B. Depletion of DNMT3A and DNMT3B, but not DNMT1 resulted in the reconstitution of STING mRNA and protein expression in B16-F10 cells (Fig. 3a, b; Supplementary Fig 4a). To directly evaluate the functional activation of STING signaling in these cells, we next stimulated them with ADU-S100. Knockdown of DNMT3A and DNMT3B significantly increased STING-dependent induction of CXCL10 (up to 2.5-fold, *p* < 0.0001) and IFN-β (up to 3.6-fold, *p* < 0.0001) in B16-F10 cells compared with siControl in response to stimulation with the STING agonist (Fig. 3c, d). Consistent with these findings, reconstitution of STING expression following pharmacologic inhibition of DNA methylation with 5AZADC coincided with decreased expression of DNMT3A and DNMT3B in B16-F10 cells (Fig. 3e). Similar effects were observed in A375 and SK-MEL-28, two STING^low^ human melanoma cell lines^37^ (Fig. 3f). Additionally, using a publicly available cBioPortal dataset^41^ we identified a negative, albeit not significant, correlation between *STING* and *DNMT3A* (Pearson’s r = −0.41), *DNMT3B* (Pearson’s r = −0.28) (Fig. 3g), as well as *DNMT1* mRNA levels (Pearson’s r = −0.13) (Supplementary Fig. 4b) across metastatic melanoma samples, suggesting their involvement in melanoma-STING silencing.

### Demethylation improves melanoma response to STING agonist therapy in STING^gt/gt^ mice

**Fig. 4:**
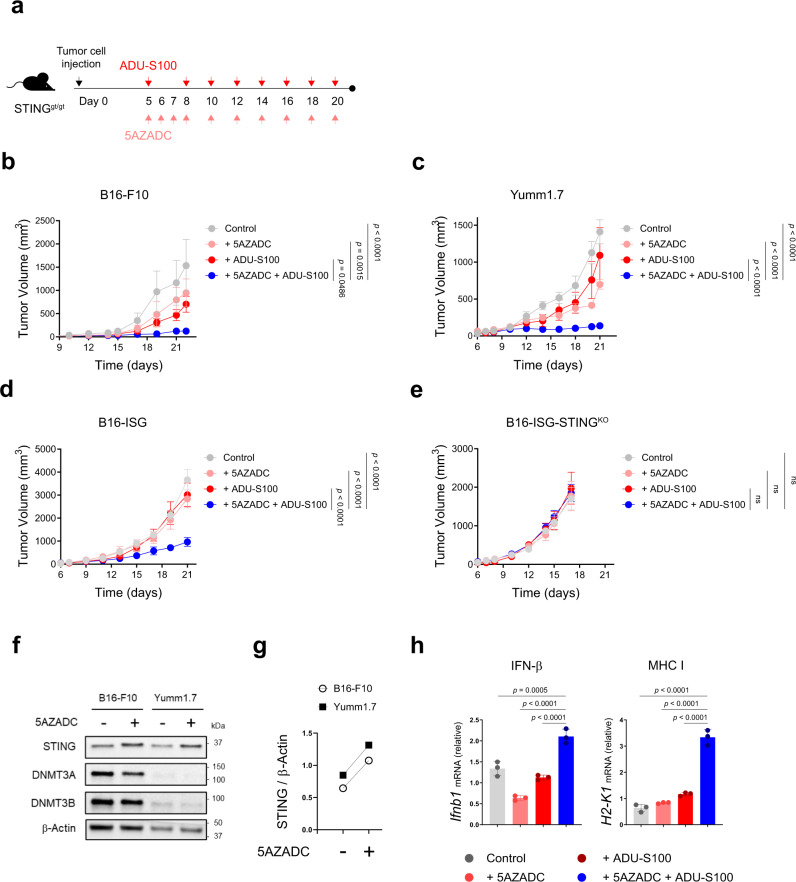
Demethylation improves melanoma response to STING agonist therapy in STING^gt/gt^ mice. Schematic of the STING agonist and 5AZADC treatment schedule. Groups of STING^gt/gt^ mice were injected subcutaneously with 1.5 × 10^5^ B16-F10 or Yumm1.7 or 1 × 10^5^ B16-ISG or B16-ISG-STING^KO^ cells on day 0 and were intratumorally treated with 50 μg of ADU-S100 and/or 0.1 mg/kg of 5AZADC or PBS (**a**). Tumor growth curves of B16-F10 (**b**), Yumm1.7 (**c**), B16-ISG (**d**) and B16-ISG-STING^KO^ (**e**) in STING^gt/gt^ mice treated with PBS, 5AZADC, ADU-S100, or 5AZADC + ADU-S100 according to the schedule presented in (**a**). Data are shown as the mean ± SEM and are representative of two independent experiments (*n* = 5 mice per group in (**b**), *n* = 4, 4, 4, and 5 mice in (**c**), *n* = 4, 6, 5, and 7 mice in (**d**), *n* = 6, 6, 5, and 6 mice in (**e**) for Control, 5AZADC, ADU-S100, and 5AZADC + ADU-S100 groups, respectively). Immunoblot analysis of STING, DNMT3A and DNMT3B expression in tumor lesions of STING^gt/gt^ mice bearing B16-F10 or Yumm1.7 tumors with or without 5AZADC treatment. β-Actin was used as a loading control (**f**). Images are representative of two independent experiments. Ratio of total STING relative to β-Actin was quantified using ImageJ version 1.53a software (**g**). Quantitative RT-PCR analysis of *Ifnb1 and H2-k1* mRNA expression in B16-F10 tumors in STING^gt/gt^ mice treated with PBS, 5AZADC, ADU-S100, or 5AZADC + ADU-S100 (n = 3 biological replicates). Data are shown as mean ± SD and are representative of two independent experiments (**h**). Statistical significance was determined by two-way (**b**–**e**) or one-way ANOVA (**h**) (ns, not significant).

To examine whether demethylation-mediated reconstitution of STING and subsequent rescue of STING signaling in STING^low^ melanoma cells could improve agonist-induced antitumor immune responses in vivo, we developed subcutaneous tumors of B16-F10 and Yumm1.7 in STING^gt/gt^ mice and treated them intratumorally with a combination of 5AZADC and ADU-S100 (Fig. 4a). While treatment with either 5AZADC or ADU-S100 alone was ineffective in delaying tumor growth, combined treatment of 5AZADC with ADU-S100 resulted in significantly enhanced inhibition of tumor growth in both B16-F10 (*p* < 0.05 on day 22) and Yumm1.7 (*p* < 0.0001 on day 21) models (Fig. 4b, c). Similarly, combination treatment with ADU-S100 and 5AZADC significantly delayed tumor growth in the aggressive model of B16-ISG (*p* < 0.0001 on day 21) (Fig. 4d); however, it failed to induce tumor control in the STING-deficient B16-ISG-STING^KO^ model (Fig. 4e) suggesting a critical role for STING in mediating antitumor responses to the combination therapy. Using immunoblot analysis, we further confirmed reconstitution of STING protein expression in B16-F10 and Yumm1.7 tumors following their in vivo treatment with 5AZADC (Fig. 4f, g). Consistent with these findings, we observed a marked increase in the transcription of *Ifnb1* (*p* < 0.0001) and *H2-k1* (*p* < 0.0001) genes downstream of STING signaling in tumors treated with the combination therapy compared to the agonist alone (Fig. 4h). Collectively, these results show that epigenetic reprogramming can restore melanoma-intrinsic STING signaling defects and therefore prime therapeutic responses to STING agonism in vivo.

### Melanoma response to combination therapy with 5AZADC and ADU-S100 depends on CD8^+^ T cells

**Fig. 5:**
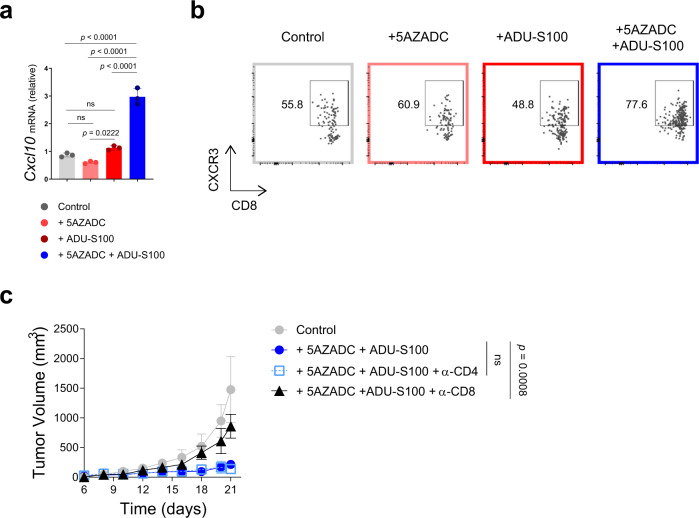
Melanoma response to combination 5AZADC and ADU-S100 therapy depends on CD8^+^ T cells. Quantitative RT-PCR analysis of *Cxcl10* mRNA expression in B16-F10 tumors in STING^gt/gt^ mice treated with PBS, 5AZADC, ADU-S100, or 5AZADC + ADU-S100 (*n* = 3 biological replicates). Data are shown as mean ± SD and are representative of two independent experiments (**a**). Representative flow cytometry plots showing frequency of CXCR3^+^ CD8^+^ T cells in B16-F10 tumors treated with PBS, 5AZADC, ADU-S100, or 5AZADC + ADU-S100. Data are representative of two independent experiments (**b**). STING^gt/gt^ mice with B16-F10 tumors were treated with PBS or 5AZADC + ADU-S100 in combination with CD8- or CD4-depleting antibodies (see Methods). Tumor growth is shown (**c)**. Data are shown as mean ± SEM (*n* = 4 mice for PBS Control, 5AZADC + ADU-S100 + α-CD4 and 5AZADC + ADU-S100 + α-CD8 and *n* = 5 mice for 5AZADC + ADU-S100 groups). Data are representative of two independent experiments (**a**–**c**). Statistical significance was determined by one-way (**a**) or two-way ANOVA (**c**). (ns, not significant).

Given the important role of CXCL10 in regulating CXCR3^+^ T cell trafficking into tumors, we next tested whether demethylation-mediated rescue of STING signaling in STING^low^ B16-F10 melanoma models could restore melanoma-intrinsic CXCL10 induction. Quantitative reverse transcription PCR analysis indicated a 2.6-fold increase (*p* < 0.0001) in *CXCL10* gene expression in B16-F10 tumors in STING^gt/gt^ mice following the combination treatment with 5AZADC and ADU-S100 (Fig. 5a). This increased CXCL10 induction correlated with higher frequency of intratumoral CXCR3^+^ CD8^+^ T cells (Fig. 5b), demonstrating that reversal of epigenetic silencing of STING using a demethylating agent can promote tumor infiltration of CD8^+^ T cells. To directly assess whether the observed antitumor effects were attributed to the generation of functional tumor antigen-specific CD8^+^ T cells, we next performed antibody-mediated depletion of either CD8^+^ or CD4^+^ T cells in B16-F10-bearing STING^gt/gt^ mice receiving the combination therapy. Successful depletions were confirmed by flow cytometry analysis of splenocytes (Supplementary Fig. 5). While depletion of CD4^+^ T cells did not alter the tumor control, depletion of CD8^+^ T cells completely abrogated the antitumor activity of the combination therapy (*p* < 0.001 on day 22) (Fig. 5c). These data indicate that CD8^+^ T cells are critical for the therapeutic efficacy of the combination therapy with 5AZADC and ADU-S100.

### CD8^+^ TILs in 5AZADC and ADU-S100 combination-treated mice indicate less exhausted phenotype

**Fig. 6:**
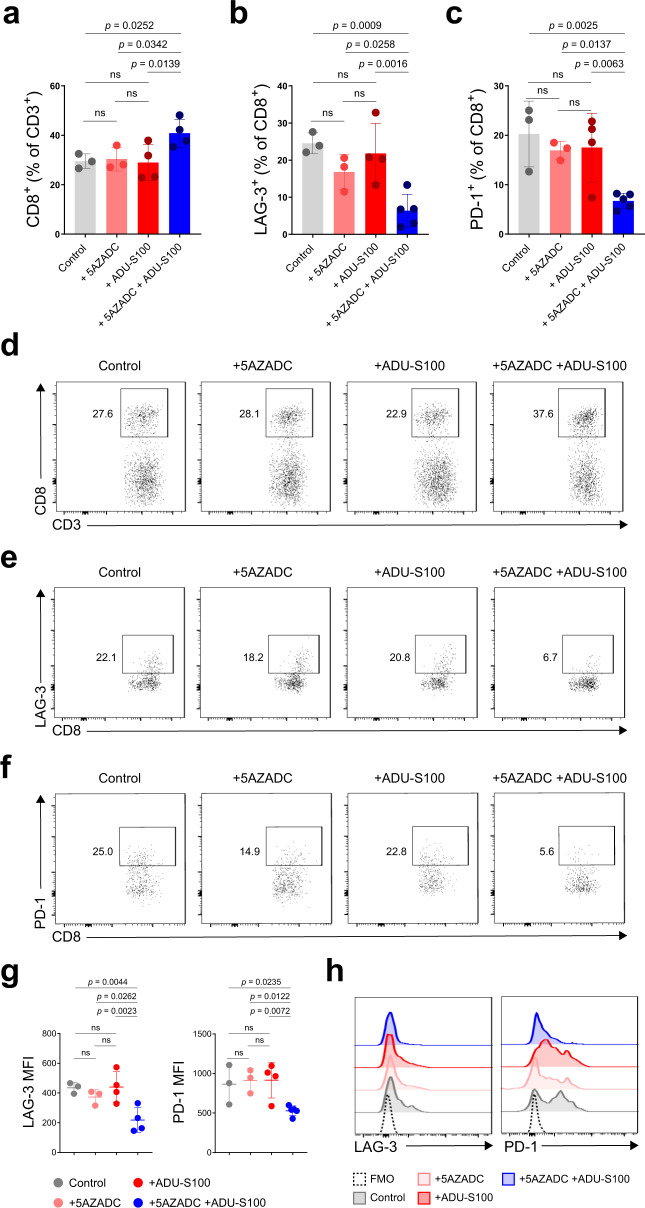
CD8^+^ TILs in 5AZADC and ADU-S100 combination therapy-treated mice indicate less exhausted phenotype. Frequency of CD8^+^ cells within the CD3^+^ population (**a**) and LAG-3^+^ (**b**) and PD-1^+^ (**c**) cells within the CD3^+^ CD8^+^ population in Yumm1.7 tumors in STING^gt/gt^ mice treated with PBS, 5AZADC, ADU-S100, or 5AZADC + ADU-S100. *n* = 3, 3, 4, and 4 mice in (**a**) and *n* = 3, 3, 4, and 5 mice in (**b**, **c**) for Control, 5AZADC, ADU-S100, and 5AZADC + ADU-S100 groups, respectively. Representative flow cytometry plots for **a**, **b**, and **c** are shown in **d**, **e**, and **f**, respectively. MFI (**g**) and representative histograms (**h**) of LAG-3^+^ and PD-1^+^ cells within the CD3^+^ CD8^+^ population in Yumm1.7 tumors in STING^gt/gt^ mice treated with PBS, 5AZADC, ADU-S100, or 5AZADC + ADU-S100. *n* = 3, 3, 4, and 4 mice for LAG-3 MFI and *n* = 3, 3, 4, and 5 mice for PD-1 MFI in (**g**) for Control, 5AZADC, ADU-S100, and 5AZADC + ADU-S100 groups, respectively. Data are representative of two independent experiments. Data are shown as the mean ± SD. Statistical significance was determined by one-way ANOVA (ns, not significant).

To characterize how combination therapy with 5AZADC and ADU-S100 can impact the tumor microenvironment, we next assessed the abundance and the phenotype of CD8^+^ TILs. Flow cytometry analysis indicated that combination treatment led to a significant increase in the frequency of tumor infiltrating CD8^+^ T cells (*p* < 0.05) (Fig. 6a, d). These CD8^+^ TIL also expressed lower levels of inhibitory receptors LAG-3 and PD-1 relative to those in the control treated tumors suggesting that combination therapy with 5AZADC and ADU-S100 can induce a functional CD8^+^ T cell population with an activated rather than an exhausted state (Fig. 6b, c, e–h). These findings, together with the observation that in the absence of CD8^+^ T cells combination therapy did not result in a therapeutic benefit (Fig. 5c), underscore the importance of CD8^+^ T cells in inducing antitumor responses in the setting of demethylation-mediated tumor cell-intrinsic STING reactivation.

### Combination therapy with 5AZADC and ADU-S100 promotes activation and effector function of CD8^+^ T cells

**Fig. 7:**
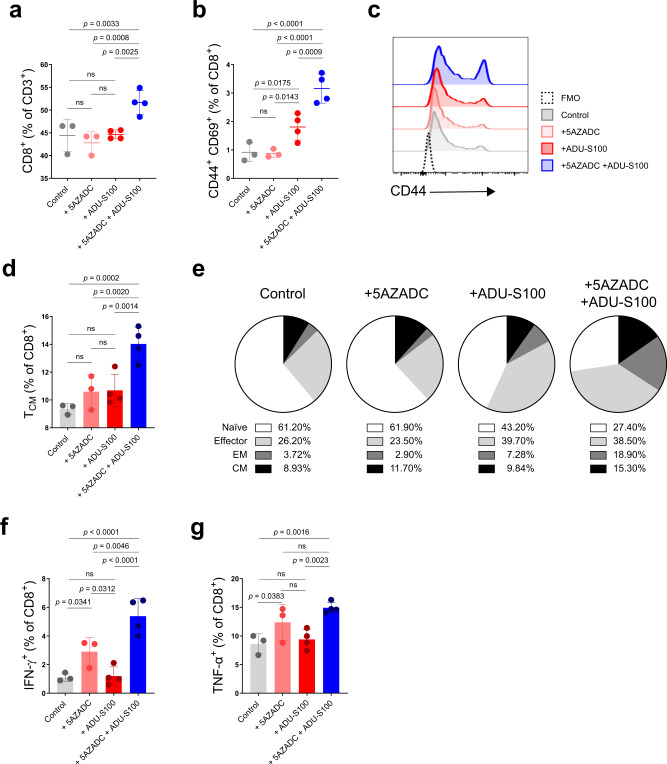
Combination therapy induces activation and effector function of splenic CD8^+^ T cells. STING^gt/gt^ mice with Yumm1.7 tumors were treated intratumorally with PBS, 5AZADC, ADU-S100, or 5AZADC + ADU-S100 as indicated in Fig. 4a; spleens were harvested on 21 after tumor cell inoculation and analyzed by flow cytometry. Shown are frequency of splenic CD8^+^ cells within the CD3^+^ population (**a**), frequency of CD69^+^ CD44^+^ cells within the CD8^+^ population (**b**), representative histograms of CD44^+^ cells within the CD3^+^ CD8^+^ population (**c**), frequency of central memory (T_CM_, CD44^+^ CD62L^+^) CD8^+^ T cells (**d**), representative pie charts indicating relative proportions of defined T cell subsets [Naïve: CD44^-^ CD62L^+^; Effector: CD44^-^ CD62L^-^; Effector Memory (EM): CD44^+^ CD62L^-^; Central Memory (CM): CD44^+^ CD62L^+^] (**e**), intracellular expression of IFN-γ (**f**) and TNF-α (**g**) in CD8^+^ T cells. Data are representative of two independent experiments. *n* = 3 mice for PBS Control and 5AZADC, and *n* = 4 mice for ADU-S100 and 5AZADC + ADU-S100 groups in (**a**–**b**), (**d**) and (**f**–**g**). Data are shown as mean ± SD. Representative flow cytometric plots for **a**, **b**, **d**, and **e**, **f**, and **g** are shown in Supplementary Fig. 6(a), (b), (c), (d), and (e), respectively. Statistical significance was determined by one-way ANOVA (ns, not significant).

To assess whether intratumoral treatment with the combination of ADU-S100 and 5AZADC induces systemic immune effects, we examined the frequency and differentiation status of T cells in the spleens of tumor-bearing mice. Within the splenic T cell population, combination treatment with 5AZADC and ADU-S100 led to an increase in the percentage of CD8^+^ T cells (Fig. 7a; Supplementary Fig. 6a). We also observed higher frequency of activated CD69^+^ CD44^+^ CD8^+^ splenic T cells in tumor-bearing mice treated with the combination of 5AZADC and ADU-S100 compared to those treated with either single agent (Fig. 7b, c; Supplementary Fig. 6b). Furthermore, the combination treatment significantly increased the central memory phenotype of CD8^+^ T cells (T_CM_, CD44^+^ CD62L^+^; *p* < 0.01) in the spleens of tumor-bearing mice (Fig. 7d, e; Supplementary Fig. 6c). Similarly, CD8^+^ T cells in tumors treated with the combination of ADU-S100 and 5AZADC indicated higher frequency of T_CM_ (Supplementary Fig. 7a, b). In addition, the activation markers CD69^+^ and CD44^+^ were expressed at higher frequency on CD8^+^ TIL from tumors treated with the combination therapy than those treated with ADU-S100 or 5AZADC alone (*p* < 0.01) (Supplementary Fig. 8a–c). We next performed intracellular cytokine staining on splenocytes from tumor-bearing mice to determine whether the combination treatment could also impact the functional state of CD8^+^ T cells. Splenic CD8^+^ T cells in tumor-bearing mice treated with the combination of 5AZADC and ADU-S100 expressed higher levels of IFN-γ and TNF-α relative to the mice treated with ADU-S100 alone (Fig. 7f, g; Supplementary Fig. 6d, e) indicating that combination therapy stimulates a stronger systemic antitumor T cell response. Overall, these results demonstrate that demethylation-mediated reactivation of STING in tumor cells can promote expansion, activation, and effector function of CD8^+^ T cells.

### Demethylation can further improve STING agonist efficacy in mice with intact STING activity

**Fig. 8:**
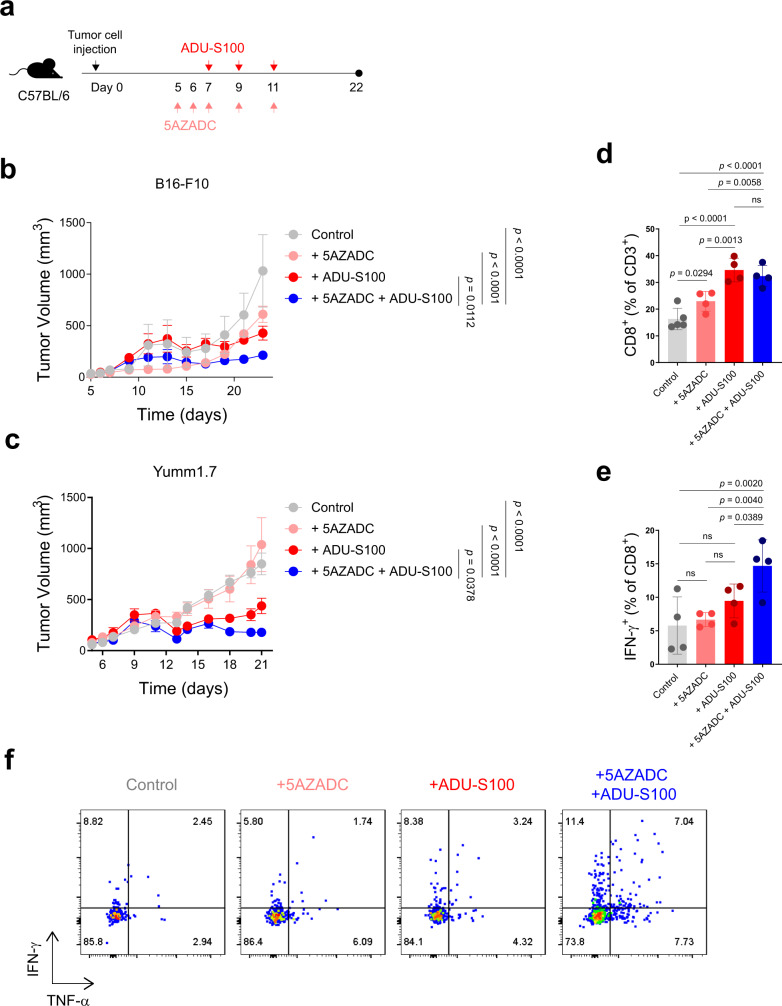
Demethylation can further improve STING agonist efficacy in C57BL/6 mice with an intact STING pathway. Schematic of the STING agonist and 5AZADC treatment schedule. Groups of C57BL/6 mice were injected subcutaneously with 1.5 × 10^5^ B16-F10 or Yumm1.7 cells on day 0 and were intratumorally treated with 50 μg of ADU-S100 and/or 0.1 mg/kg of 5AZADC or PBS (**a**). Tumor growth curves of B16-F10 (**b**) and Yumm1.7 (**c**) in C57BL/6 mice intratumorally treated with PBS, 5AZADC, ADU-S100, or 5AZADC + ADU-S100 according to the schedule presented in (**a**). Data are shown as the mean ± SEM (**b**–**c**). Control, *n* = 3; 5AZADC, *n* = 4; ADU-S100, *n* = 6; and 5AZADC + ADU-S100, *n* = 7 mice in (**b**) and Control, *n* = 6; 5AZADC, *n* = 5; ADU-S100, *n* = 5; and 5AZADC + ADU-S100, *n* = 5 mice in (**c**). Frequency of CD8^+^ T cells (**d**) and IFN-γ expressing CD8^+^ T cells (**e**) in Yumm1.7 tumors in C57BL/6 mice treated with PBS, 5AZADC, ADU-S100, or 5AZADC + ADU-S100 on day 21. *n* = 5, 4, 4, 4 mice in (**d**) and *n* = 4, 4, 4, 4 mice in (**e**) for Control, 5AZADC, ADU-S100, and 5AZADC + ADU-S100 groups, respectively. Data are shown as mean ± SD. Representative flow cytometry plots of intracellular cytokine staining for IFN-γ and TNF-α in CD8^+^ T cells in Yumm1.7 tumors in C57BL/6 mice treated with PBS, 5AZADC, ADU-S100, or 5AZADC + ADU-S100 on day 21 (**f**). Data are representative of two independent experiments. Statistical significance was determined by two-way (**b**–**c**) or one-way ANOVA (**d**–**e**) (ns, not significant).

To further investigate the functional importance of demethylation-induced STING reactivation in tumor cells in enhancing antitumor responses to STING agonist therapy in a host with intact STING activity, we injected C57BL/6 mice with B16-F10 or Yumm1.7 tumor cells and treated them with a combination of ADU-S100 and 5AZADC (Fig. 8a). In this setting, where STING is functional in the host antigen presenting cells, ADU-S100 alone resulted in tumor control compared to 5AZADC or vehicle treated controls; however, combination therapy was more effective in inhibiting the tumor growth in both B16-F10 and Yumm1.7 models (Fig. 8b, c). Although there was no detectable difference in the percentage of CD8^+^ TIL (Fig. 8d), tumors treated with a combination of ADU-S100 and 5AZADC were more enriched with effector cytokine expressing CD8^+^ T cells than those treated with ADU-S100 alone (Fig. 8e, f). These data show that restoration of STING activity in tumor cells can augment antitumor responses to STING agonist therapy even in STING-intact hosts and indicate that both tumor and host-derived STING, together, can play a critical role in promoting antitumor immunity.

## Discussion

Direct activation of the host STING pathway through intratumoral injections of synthetic cyclic dinucleotides has shown to be an attractive therapeutic strategy in multiple preclinical cancer models^22,25,42^. However, clinical trials in cancer patients currently testing STING activating drugs as a monotherapy or in combination with immune checkpoint inhibitors have so far provided little, if any, evidence of objective responses^26–28^.

This discrepancy in outcomes to STING agonist therapies raises the possibility that targeting the STING pathway could be functionally relevant not only in antigen presenting cells of the host, which has been the focus of prior preclinical studies, but also in tumor cells with well documented epigenetically driven dysfunctional STING activity across several human cancer types, including melanoma^29,35,43,44^. Despite this common suppression, the ability to target and restore STING signaling in tumor cells using epigenetic modulators^35,37^ provides a rationale to explore whether epigenetic reprogramming of tumor cell-intrinsic STING activity can improve the therapeutic efficacy of STING agonists. In this study, we investigated this rationale in vivo using multiple approaches with murine melanoma models in both STING-deficient and STING-sufficient hosts.

Consistent with our previous findings in human melanoma^37^, we found promoter hypermethylation-driven STING silencing in two distinct murine melanoma cell lines. Both in vitro and in vivo, pharmacologic inhibition of DNA methylation with 5AZADC resulted in reconstitution of STING protein expression indicating the involvement of common epigenetic mechanisms in regulating STING expression in these experimental models. We further showed that demethylation-mediated reversal of STING silencing not only greatly increased agonist-induced production of IFN-β by tumor cells in in vitro experiments, but also within the tumors in STING-deficient mice. In host dendritic cells, STING-mediated IFN-β induction has been well characterized and shown to be essential for their activation and the cross-priming of cytotoxic T cells^22,23^. Similarly, initiation of an adaptive immune response to radiation therapy has been linked to STING-mediated IFN-β induction by dendritic cells^45^. Nevertheless, compelling clinical and experimental evidence support the importance of IFN-β production by tumor cells themselves in mediating antitumor responses to radiation therapy in combination with immune checkpoint inhibitors^46–48^. In agreement with these studies, we found that demethylation-mediated restoration of STING-dependent IFN-β production in tumors resulted in greater tumor control. IFN-β induction in tumor cells can exert direct effects both on tumor cells as well as APCs^16,47^. Indeed, we have previously shown that in human melanoma cell lines STING-mediated IFN-β induction can augment their surface expression of MHC class I and therefore render them better targets for cytotoxic CD8^+^ T cell recognition and killing^29,37^. The importance of IFN-β production by cancer cells in the recruitment and activation of Batf3-dependent dendritic cells has been also demonstrated in different settings^47,49^. Although mechanistically it remains unclear, it is conceivable that demethylation-mediated restoration of STING-dependent IFN-β production in tumors can take a more active and direct role in driving the maturation and activation of tumor-infiltrating dendritic cells.

Similar to our observations in human melanoma cell lines, CXCL10 was upregulated in mouse melanoma tumors following demethylation-mediated reactivation of STING signaling. Together with CXCL9, another CXCR3-binding chemokine, CXCL10 is a dominant chemokine for the recruitment of tumor-specific CD8^+^ T cells into the tumors^31,32^. Also, as a member of the 12-chemokine gene signature classifier^33^, its expression has been correlated with the presence of tumor-associated tertiary lymphoid structures^50–52^ which are rapidly emerging as a powerful prognostic marker in melanoma and a number of other cancer types^53–55^. In our studies, tumor-localized CXCL10 induction was correlated with higher frequency of CD8^+^ T cells within tumors. These data suggest that STING-functional tumors can initiate a CXCL10/CXCR3 axis and subsequently guide trafficking and entry of effector T cells into the tumor sites. Additionally, CXCL10 has been shown to promote effector T cell generation and function^56,57^. In line with these data, we found a CD8^+^ T cell-dependent mechanism in mediating antitumor effects in response to the combination therapy. This finding also coincides with our observation of STING-dependent MHC class I upregulation in human and mouse melanoma cells following demethylation-mediated restoration of STING signaling which can enhance both their antigenicity and susceptibility to lysis by CD8^+^ T cells^29,37^. Although it was proposed that STING agonist can induce T cell death through the activation of proapoptotic transcriptional programs^58^, recent studies have indicated that this effect depends on the type and doses of STING agonists^59,60^. Unlike the mouse STING ligand DMXAA that was shown to trigger T cell death, stimulation of T cells with cyclic dinucleotides including cGAMP did not cause T cell death. Also, two recent reports have shown that T cell-intrinsic STING signaling could control differentiation and effector functions of CD8^+^ and CD4^+^ T cells^60,61^. Although our initial in vivo studies were performed in STING^gt/gt^ mice with T cells lacking STING and therefore insensitive to STING agonist, our later experiments in STING-sufficient C57/BL6 mice did not indicate any unfavorable effect on the frequency or function of T cells of the mice treated with either ADU-S100 alone, or when combined with 5AZADC.

Furthermore, consistent with mobilization and expansion of CD8^+^ T cells within the tumors, we found an increase in the frequency of splenic CD8^+^ T cells in tumor-bearing mice treated with intratumoral injections of ADU-S100 and 5AZADC. These splenic CD8^+^ T cells mainly displayed an effector/memory phenotype as indicated by higher expression of antigen-experience and activation markers including CD44 and CD69, as well as higher frequency of IFN-γ effector cytokine producing cells. Based on these observations, it is likely that local tumor cell-induced type I IFN response driven by the rescue and activation of STING signaling can promote a systemic antitumor CD8^+^ T cell immunity. This notion is further supported by two recent publications describing a novel role for cancer cells and their type I IFN production in priming CD8^+^ T cell responses through MHC class I dressing of dendritic cells^49,62^. Although additional experiments will be needed to fully understand the factors contributing to the systemic activation that we observed, regardless of the exact mechanism, our data indicate that targeting tumor-cell intrinsic STING signaling through intratumoral combination therapy is capable of priming antitumor CD8^+^ T cell responses.

As restoration and reactivation of STING pathway in tumor cells can reinstate their antigenicity as well as T cell tumor trafficking, a key question for future work will be if this strategy could improve the efficacy of T cell-based immunotherapies in patients with melanoma particularly those involving adoptive cell transfer of autologous TIL. Similarly, it will be important in the future to determine how targeting tumor cell-intrinsic STING activity in solid tumors beyond melanoma can affect immunotherapy outcomes. Restoration of tumor cell-intrinsic STING activity through epigenetic remodeling may be also beneficial for CAR T-cell therapies by modulating the tumor microenvironment and providing a favorable milieu for CAR T-cell trafficking and persistence in solid tumors^63^.

In summary, our data demonstrate that epigenetic silencing of STING in melanoma cells is not only a marker of tumor-intrinsic immune evasion mechanism but can indeed confer resistance to STING agonist therapy. Accordingly, we have shown that a rational combination of a clinically available DNA methylation inhibitor with a STING agonist can lead to robust antitumor responses in the setting of STING^low^ murine models of melanoma. Therefore, identification and pharmacologic restoration of tumor cell-intrinsic STING signaling defects through epigenetic reprograming might be critical for the successful use of STING agonist therapies in the clinic. Although additional work will be necessary to further identify optimal dosing levels and therapeutic schedules of each component, insights from our study provide a framework to design proper clinical treatment modalities with appropriate patient selection in melanoma and perhaps other solid tumors.

## Methods

### Melanoma cell lines

A375 and SK-MEL-28 (provided by Dr. Keiran Smalley, Moffitt Cancer Center); B16-ISG and B16-ISG-STING^KO^ (purchased from InvivoGen) were cultured in RPMI 1640 (Thermo Fisher Scientific). B16-F10 [purchased from American Type Culture Collection (ATCC)] and Yumm1.7 (provided by Dr. Keiran Smalley, Moffitt Cancer Center) were cultured in Dulbecco’s modified Eagle’s medium (DMEM; Thermo Fisher Scientific). In all cases, media contained 10% heat-inactivated fetal bovine serum (FBS) (Omega Scientific) and antibiotics. Growth medium for B16-ISG and B16-ISG-STING^KO^ was also supplemented with 100 μg/ml of Zeocin (InvivoGen). Accell Small interfering RNA (siRNA) smart pool against mouse DNMT1 (catalog no. E-056796-00-0020; accession no. NM_001199431, NM_001199432, NM_001199433, NM_001314011, NM_010066, XM_006509988, and XM_011242393), DNMT3A (catalog no. E-065433-00-0020; accession no. NM_001271753, NM_007872, NM_153743, XM_006514953, and XM_006514956) and DNMT3B (catalog no. E-044164-00-0020; accession no. NM_001003960, NM_001003961, NM_001003963, NM_001122997, NM_001271744, NM_001271745, NM_001271746, NM_001271747, NM_010068, XM_006498682, XM_006498683, XM_006498684, XM_006498685, XM_006498686, XM_006498687, XM_006498688, XM_006498689, and XR_374399) and non-targeting control pool (catalog no. D-001910-10-50) were obtained from Dharmacon. The siRNAs were diluted in the supplied 5XsiRNA buffer diluted in RNase-free water and used for the transfection of B16-F10 cells according to the manufacturer′s instructions. Cells were subjected to further analyses following 48 h incubation at 37 °C. Genomic DNA for B16-F10 and Yumm1.7 cell lines was extracted using the Blood & Cell Culture DNA Mini Kit (Qiagen), according to the manufacturer’s instructions for cultured cells.

### Analysis of methylation data

The Infinium Mouse Methylation BeadChip was processed and normalized with GenomeStudio (V2011.1 Illumina Inc.) using control probes and background subtraction^64^. The β-values and corresponding p-values was exported to MATLAB (R2020a, Natick, MA, USA) for further QC and analysis. The visualization of methylation data was performed in GraphPad Prism.

### Database analysis

RNA-seq data of STING and individual DNMTs (DNMT1, DNMT3A and DNMT3B) in a melanoma dataset^41^ were downloaded from cBioPortal (http://www.cbioportal.org) on January 17, 2022, and exported to GraphPad Prism for visualization and calculation of Pearson’s correlation coefficient.

### Mice

All animal experiments were developed under an Institutional Animal Care and Use Committee (IACUC) protocol (IS00009850) approved by the Integrity and Compliance board at the University of South Florida and Moffitt Cancer Center in accordance with the U.S. Public Health Service policy and National Research Council guidelines. Wild-type C57BL/6 (#000664) and STING^gt/gt^ (Goldenticket; C57BL/6J-Sting1^gt/^J; #017537) mice were obtained from The Jackson Laboratory. All experiments were initiated using female mice between the ages of 8 and 10 weeks. Animals were housed in the Comparative Medicine Facilities at the Moffitt Cancer Center under temperature and humidity-controlled conditions with a 12-h light/dark cycle. At experimental endpoints, mice were humanely euthanized using CO_2_ inhalation followed by cervical dislocation in accordance with American Veterinary Medical Association guidelines.

### In vivo tumor models and treatment

On day 0, mice were injected with 1 × 10^5^ to 2 × 10^5^ tumor cells subcutaneously in the back. Five days after tumor inoculation, tumor-bearing STING^gt/gt^ mice were treated with a 100-μl complex containing 50 μg of ADU-S100 (chemietek) and/or 0.1 mg/kg of 5AZADC (Sigma-Aldrich) or PBS (as vehicle control) intratumorally (I.T.) as described previously^25^. For tumor-bearing STING^gt/gt^ mice, the I.T. injection was performed every 2–3 days thereafter for the duration of the study. Tumor-bearing C57BL/6 mice were treated I.T. with 50 μg of ADU-S100 on days 7, 9, and 11 and/or 0.1 mg/kg of 5AZADC on days 5, 6, 7, 9, and 11, with control groups receiving I.T. injection of PBS. Tumors were measured every 2 days with a caliper. Tumor volume was determined using the formula: (small diameter)^2^ ×  (large diameter) × 0.5. To deplete T cells, mice were injected intraperitoneally with 300 μg of anti-CD8 (clone 2.43, catalog no. BE0061, Bio X Cell), or anti-CD4 (clone GK1.5, catalog no. BE0003-1, Bio X Cell) 5 days prior to tumor implantation, and every 2–3 days thereafter for the duration of the study. Cellular depletions were confirmed by flow cytometry of splenocytes (Supplementary Fig. 5).

### In vitro 5AZADC treatment

Human and mouse melanoma cell lines were treated with 0.1–1 μM 5AZADC (Sigma Aldrich) dissolved in culture medium that was prepared and replaced daily. At day 3, cells were washed and replenished with fresh culture medium (without 5AZADC) and rested for an additional 3 days before assaying (day 6). For the IFNAR blocking studies, mouse melanoma cell lines were treated with 5AZADC in the presence of anti-IFNAR1 (clone MAR1-5A3, catalog no. 16-5945-85, Invitrogen) at a final concentration of 10 μg/mL.

### In vitro stimulation with STING agonist

Mouse melanoma cell lines (4 × 10^5^ cells/well in 24-well plates) were exposed to the STING agonist ADU-S100 (10 µg/ml) in the presence of Lipofectamine 2000 (Invitrogen) according to the manufacturer’s instructions as previously described^29^. After 24 h of incubation at 37 °C in a humidified CO_2_ incubator, the supernatants were collected for detection of IFN-β and CXCL10 release using enzyme-linked immunosorbent assays (DuoSet ELISA Kits, R&D Systems).

### Immunoblot analysis

Proteins were extracted with RIPA buffer (Thermo Fisher Scientific) containing protease inhibitors (Thermo Scientific). Equal amounts of proteins were resolved on SDS-PAGE gels (Bio-Rad) and transferred to polyvinylidene fluoride (PVDF) membranes (Bio-Rad). After blocking with 5% non-fat dry milk, membranes were incubated with the following antibodies (clone, dilution, supplier, catalog no.): STING (D2P2F, 1:1000, Cell Signaling, 13647S), DNMT1 (D63A6, 1:1000, Cell Signaling, 5032S), DNMT3A (D23G1, 1:1000, Cell Signaling, 3598S), DNMT3B (D7O7O, 1:1000, Cell Signaling, 67259S), LMP2 (EPR22042, 1:1000, Abcam, ab3328), α-Tubulin (DM1A, 1:5000, Cell Signaling, 3873S), and β-Actin (AC-74, 1:5000, Sigma-Aldrich, A5316). Following incubation with appropriate secondary antibodies [anti-rabbit (dilution 1:2000, Cell Signaling, catalog no. 7074S), anti-mouse (dilution 1:2000, Cell Signaling, catalog no. 7076S)], bands were visualized using an enhanced chemiluminescence detection system.

### Flow cytometry

Single-cell suspensions of spleens were generated by passing cells through a 40-μm cell strainer. Red blood cells (RBCs) were removed from spleens using RBC lysis buffer (BioLegend). Tumor cell suspensions were prepared by enzymatic digestion in Hanks’ Balanced Salt Solution (HBSS; Life Technologies) containing 1 mg/ml collagenase IV, 0.1 mg/ml DNaseI, and 2.5 U/ml hyaluronidase (all from Sigma-Aldrich) and then subjected to GentleMACS dissociation (Miltenyi Biotec). Tumor digest cell suspensions were incubated at 37 °C in a rocking water bath for 1 h. RBCs were removed using RBC Lysis Buffer (BioLegend), then cell suspensions were filtered with a 100 μm cell strainer to remove large cellular debris. All cells were stained with LIVE/DEAD Fixable Dead Cell Stain (Life Technologies) to distinguish live from dead cells. To prevent nonspecific antibody binding, cells were preincubated with anti-CD16/CD32 antibody (dilution 1:50, BD Biosciences, catalog no. 553142) for 10 minutes before cell surface staining. Cell surface staining was performed for 20 min at 4 °C with the following antibodies (clone, dilution, supplier, catalog no.): anti-mouse CD3e BUV395 (145-2C11, 1:50, BD Biosciences, 565533), CD4 BV786 (GK1.5, 1:100, BD Biosciences, 563331), CD69 PE-CF594 (H1.2F3, 1:100, BD Biosciences, 562455), and CD279 (PD-1) BV605 (J43, 1:50, BD Biosciences, 563059), CD8a PE/Dazzle 594 (53-6.7, 1:200, Biolegend, 100762), CD8a Alexa Fluor 700 (53-6.7, 1:200, Biolegend, 100730), CD183 BV421 (CXCR3-173, 1:200, BD Biosciences, 566283), CD45 BB515 (30-F11, 1:200, BD Biosciences, 564590), CD62L PE/Cy7 (MEL-14, 1:100, Biolegend, 104418), CD44 Alexa Fluor 488 (IM7, 1:200, Biolegend, 103016), H-2Kb Alexa Fluor 647 (AF6-88.5, 1:100, Biolegend, 116512), and LAG3 BV421 (C9B7W, 1:200, Biolegend, 125221). For intracellular cytokine staining, cells were first incubated for 5 h at 37 °C in the presence of GolgiPlug (BD Biosciences). Cells were then fixed and permeabilized using Cytofix/Cytoperm buffer (BD Biosciences) and stained with anti-mouse IFN-γ PE (XMG1.2, 1:10, BD Biosciences, 554412) and TNF-α BV650 (MP6-XT22, 1:10, BD Biosciences, 563943) for 30 min at 4 °C in Perm/Wash buffer (BD Biosciences). Flow cytometry sample acquisition was performed on an LSR II cytometer (BD Biosciences) with BD FACSDiva version 9 (BD Biosciences), and the data were analyzed using FlowJo version 10.7.1 software (TreeStar).

### RNA isolation, reverse transcription, and quantitative polymerase chain reaction

Tumors were mechanically dissociated and lysed with RLT buffer. Total cellular RNA was extracted using the RNeasy Mini Kit (Qiagen) according to manufacturer’s instructions. Following extraction, 0.5 to 1 μg total RNA was used to generate Complementary DNA (cDNA) using the iScript reverse transcription kit (Bio-Rad). Quantitative real-time polymerase chain reaction (PCR) of the indicated genes was performed using SsoFast EvaGreen Supermix (Bio-Rad) in a CFX96 Thermocycler (Bio-Rad). Relative expression was calculated using the ΔΔCt method and normalized to *Actb* levels. Primers for murine *STING* (unique assay ID: qMmuCID0016081), *Ifnb1* (unique assay ID: qMmuCED0050444), *Cxcl10* (unique assay ID: qMmuCED0049500), *H2-k1* (unique assay ID: qMmuCED0004490), *Tap1* (unique assay ID: qMmuCID0005233), and *Actb* (unique assay ID: qMmuCED0027505) were purchased from Bio-Rad.

### Statistical methods

Statistical analyses were performed using GraphPad Prism 9 (GraphPad Software) as previously described^29^. All data are presented as mean ± SD unless otherwise indicated. In all cases, unpaired two-tailed Student t test for comparisons of two groups or one-way or two-way analysis of variance (ANOVA) for comparisons of multiple groups were used as described in the figure legends. Significance was defined as follows: *p < 0.05; **p < 0.01; ***p < 0.001; and ****p < 0.0001.

### Reporting summary

Further information on research design is available in the Nature Portfolio Reporting Summary linked to this article.

## Supplementary information

Supplementary Information

Reporting Summary

## Data Availability

All study data are included in the article and/or supplementary information files. Genomic data are from the cBioPortal repository (https://www.cbioportal.org/study/summary?id=skcm_mskcc_2014). Source data are provided with this paper.

## Source data

Source Data
