# Supplementary material for: Epigenetic state determines the in vivo efficacy of STING agonist therapy
Source: Nat Commun. 2023 Mar 22;14:1573. doi: 10.1038/s41467-023-37217-1 (PMC10033671; doi:10.1038/s41467-023-37217-1)
Supplement: Supplementary file 1 — Supplementary Information [file 41467_2023_37217_MOESM1_ESM.pdf]

Epigenetic state determines the *in vivo* efficacy of STING agonist therapy

Rana Falahat[1], Anders Berglund[2], Patricio Perez-Villarroel[1], Ryan M. Putney[2], Imene Hamaidi[1], Sungjune Kim[1,3], Shari Pilon-Thomas[1,4], Glen N. Barber[5], and James J. Mulé[1,3,4]*

[1]Department of Immunology, Moffitt Cancer Center; Tampa, FL 33612
[2]Department of Biostatistics and Bioinformatics, Moffitt Cancer Center; Tampa, FL 33612
[3]Radiation Oncology Program, Moffitt Cancer Center; Tampa, FL 33612
[4]Cutaneous Oncology Program, Moffitt Cancer Center; Tampa, FL 33612
[5]Department of Cell Biology, University of Miami Miller School of Medicine; Miami, FL 33136

*Corresponding Author: James J. Mulé, Moffitt Cancer Center, 12902 Magnolia Drive, Tampa, FL 33612. Phone: 813-745-1536; Fax: 813-745-6188; E-mail: James.Mule@moffitt.org

Supplementary Figure 1

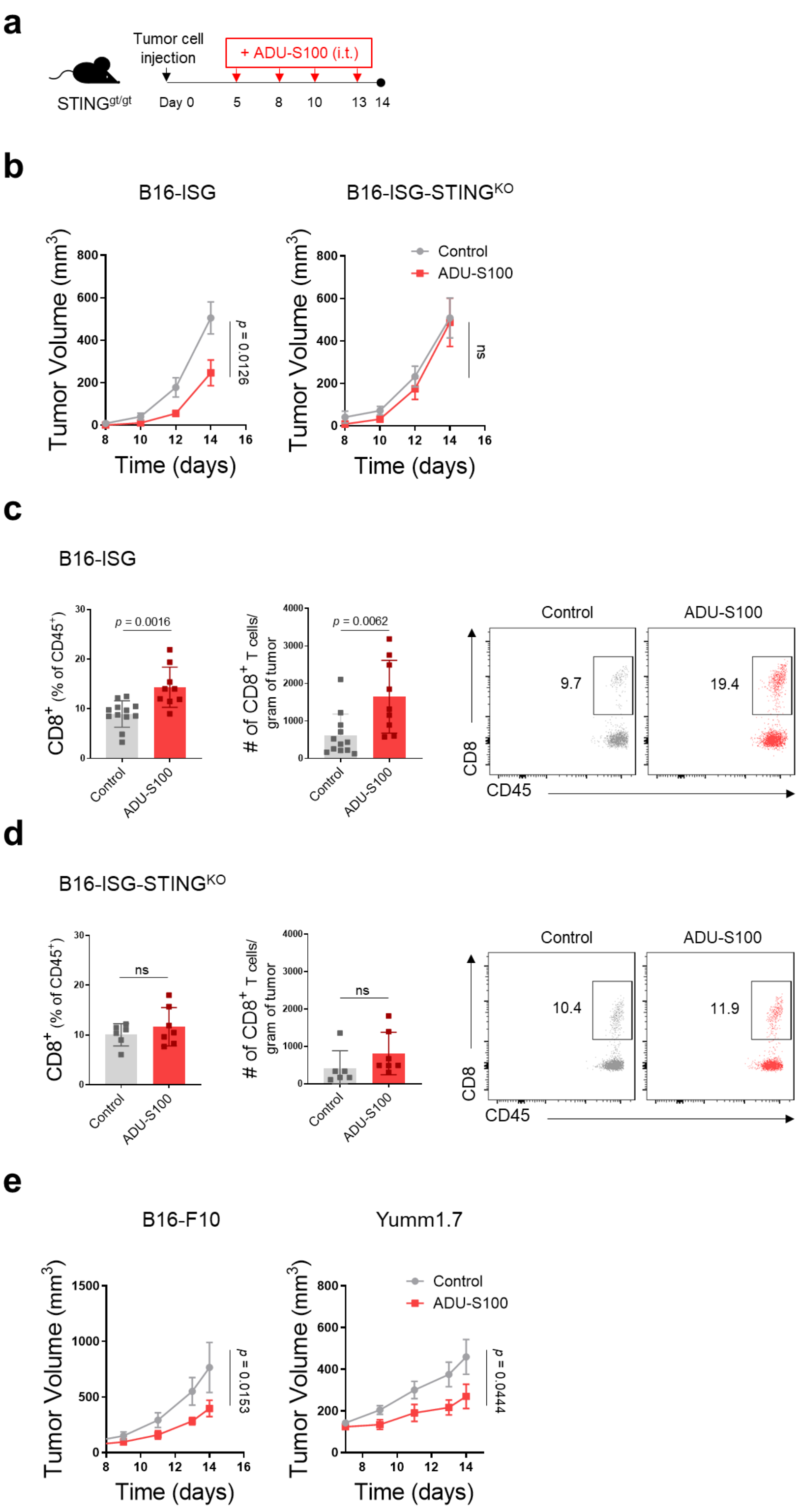

Supplementary Figure 1. Melanoma-intrinsic STING activation results in delayed tumor growth and increased tumor-infiltrating CD8$^+$ T cells in the low tumor burden state.

Schematic of the STING agonist treatment schedule. Groups of STING$^{gt/gt}$ mice were injected subcutaneously with 1 x 10^5 B16-ISG or B16-ISG-STING^{KO} or 1.5 x 10^5 B16-F10 or Yumm1.7 on day 0. After tumor injection, tumor-bearing mice were intratumorally treated with either PBS or ADU-S100 (50 μg) on days 5, 8, 10, and 13 **(a)**. Average tumor volume of B16-ISG and B16-ISG-STING^{KO}–bearing STING$^{gt/gt}$ mice treated with PBS control or ADU-S100 as indicated in (a). Data are shown as mean ± SEM. Data are representative of three independent experiments, B16-ISG (n = 11 and 14 mice for Control and ADU-S100 groups, respectively), B16-ISG-STING^{KO} (n = 5 and 6 mice for Control and ADU-S100 groups, respectively) **(b)**. Frequency (left), total number (middle), and representative flow cytometry plots (right) of CD8$^+$ cells within the CD45$^+$ population in B16-ISG (n = 12 and 9 mice for Control and ADU-S100 groups, respectively) and B16-ISG-STING^{KO} (n = 6 and 7 mice for Control and ADU-S100 groups, respectively) tumors treated with PBS control or ADU-S100. Data are shown as mean ± SD **(c-d)**. B16-F10 and Yumm1.7 tumor growth in STING$^{gt/gt}$ mice treated with PBS control or ADU-S100 as indicated in (a). Data are shown as mean ± SEM. Data are representative of two independent experiments, B16-F10 (n = 7 mice per group), Yumm1.7 (n = 6 mice per group) (**e**). Statistical significance was determined by Unpaired Student’s t-test (b-e) (ns, not significant).

Supplementary Figure 2

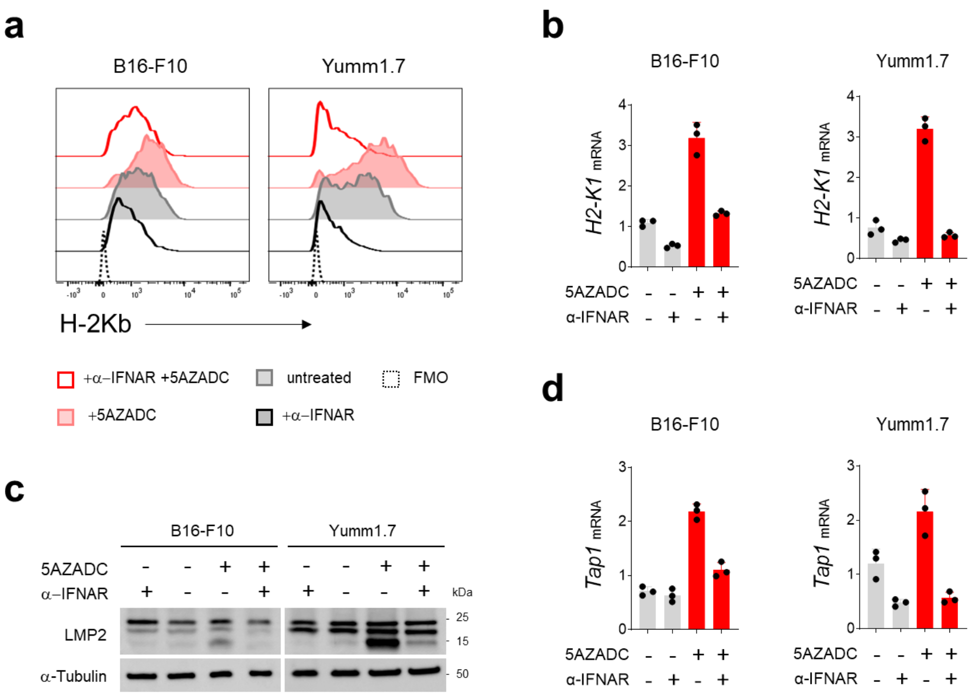

Supplementary Figure 2. 5AZADC-induced upregulation of MHC I depends on type I IFN signaling.

B16-F10 and Yumm1.7 cells were treated with 5AZADC in the presence or absence of an IFNAR blocking antibody. Representative histograms of MHC I (H2-Kb) expression **(a)**, quantitative RT-PCR analysis of *H2-k1* mRNA expression **(b)**, immunoblot analysis of LMP2 **(c)**, and *Tap1* mRNA expression **(d)** on indicated cells. Data are shown as mean ± SD of three biological replicates and are representative of two independent experiments.

Supplementary Figure 3

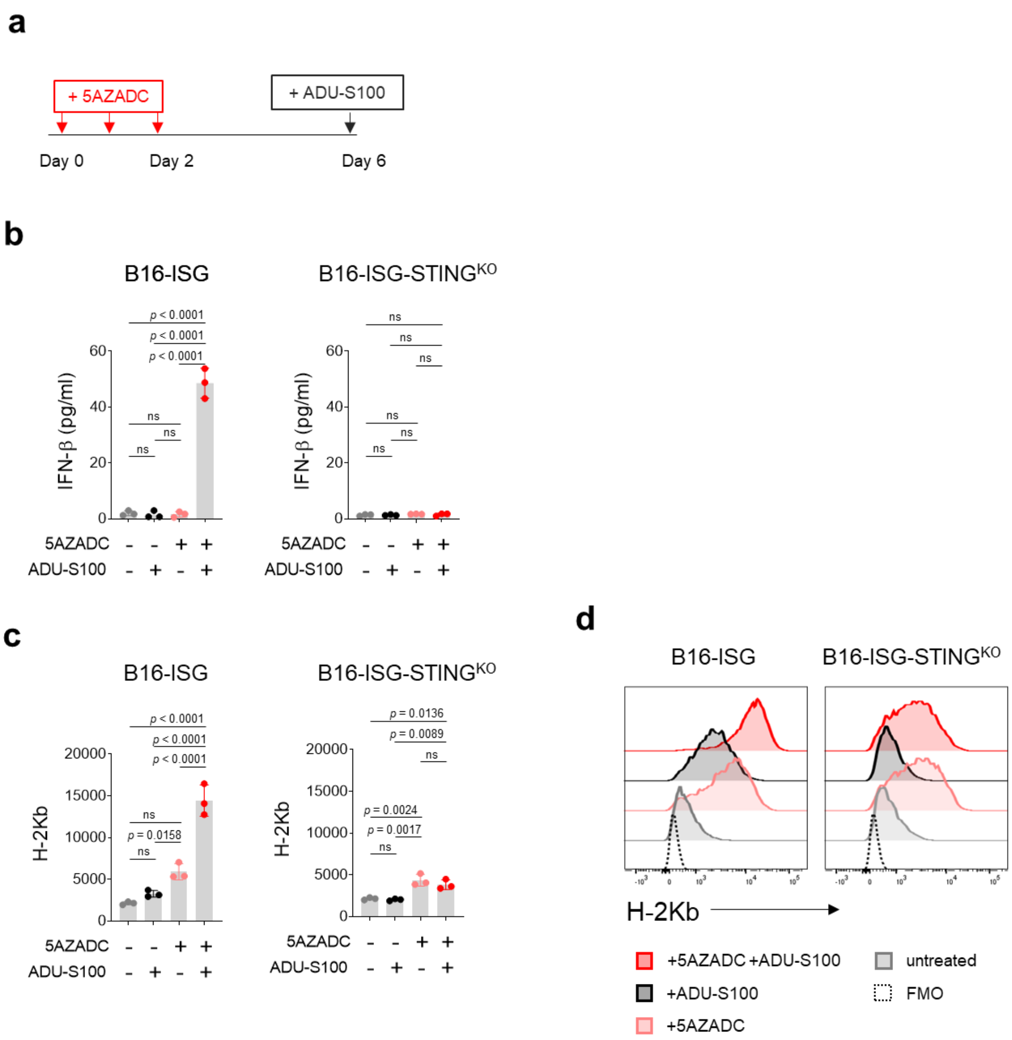

Supplementary Figure 3. DNA demethylation-induced increased IFN-β production and upregulation of MHC I in response to agonist stimulation in melanoma cells is STING mediated.

Experimental setup for 5AZADC treatment of B16-ISG and B16-ISG-STING^{KO} cell lines **(a)**. Following 5AZADC treatment, melanoma cells were stimulated with the STING agonist ADU-S100 for 24 h. Induction of IFN-β in cell culture supernatants measured using ELISA **(b)**. Mean fluorescence intensity (MFI) **(c)** and representative histograms of MHC I (H2-Kb) expression **(d)** on indicated cell lines. Statistical significance was determined by one-way ANOVA (b and c) (ns, not significant). Data are shown as mean ± SD of three biological replicates and are representative of two independent experiments.

Supplementary Figure 4

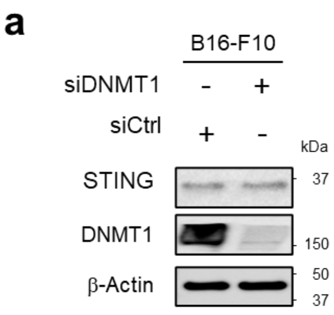

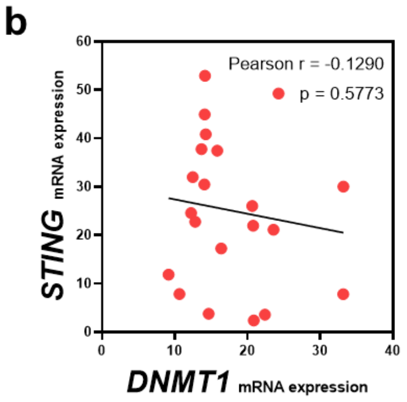

Supplementary Figure 4. DNMT1 expression does not correlate with STING silencing in melanoma.

Immunoblot analysis of STING and DNMT1 expression in B16-F10 cells transfected with siRNA specific for DNMT1 (siDNMT1) or nontarget siRNA (siControl). β-Actin was used as a loading control **(a)**. Images are representative of three independent experiments. Correlative analysis of *STING* mRNA expression with *DNMT1* in metastatic melanoma samples (n=21) from cBioPortal database **(b)**.

Supplementary Figure 5

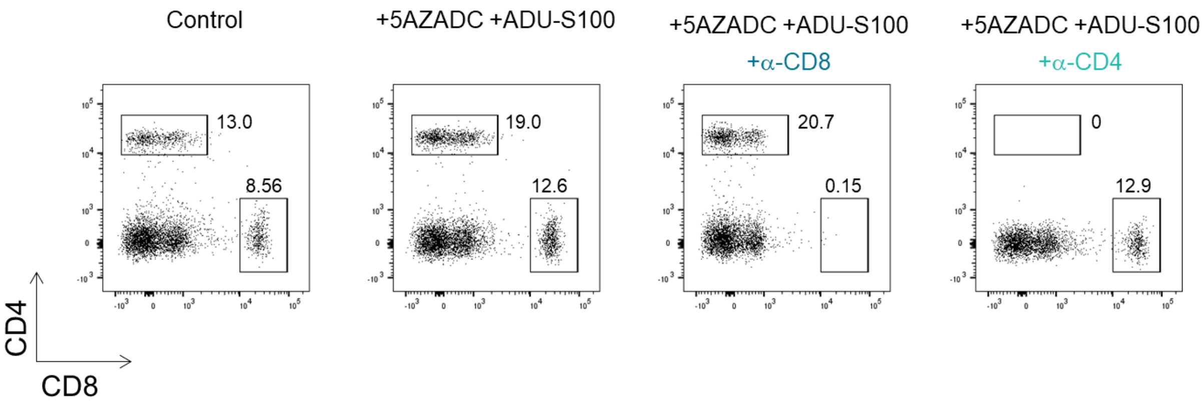

Supplementary Figure 5. Verifying *in vivo* depletions.

Confirmation of depletions for $CD8^+$ T cells and $CD4^+$ T cells in splenocytes. To deplete T cells, mice were injected intraperitoneally with 300 μg of anti-CD8 (clone 2.43, Bio X Cell), or anti-CD4 (clone GK1.5, Bio X Cell) 5 days prior to tumor implantation, and every 2-3 days thereafter for the duration of the study. Representative flow cytometry plots indicating expression of CD4 and CD8 in $CD45^+$ $CD3^+$ splenic T cells on day 21.

Supplementary Figure 6

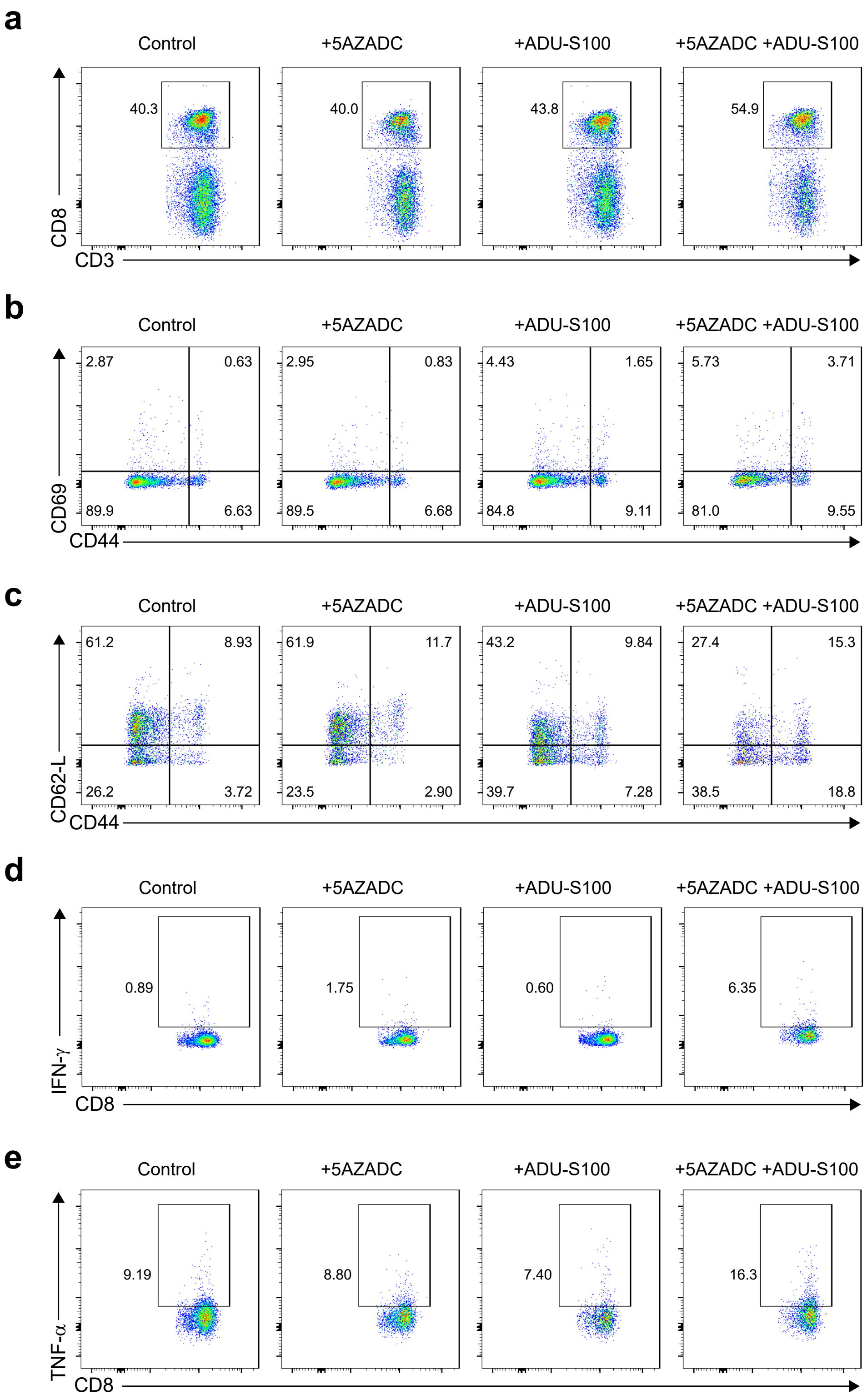

Supplementary Figure 6. Representative flow cytometry plots for Figure 7.

$STING^{gt/gt}$ mice with Yumm1.7 tumors were treated intratumorally with PBS, 5AZADC, ADU-S100, or 5AZADC+ADU-S100 as indicated in Figure 4a; spleens were harvested on 21 after tumor cell inoculation and analyzed by flow cytometry. Shown are representative flow cytometry plots indicating frequency of splenic $CD8^+$ cells within the $CD3^+$ population **(a)**, frequency of $CD44^+$ $CD69^+$ cells within the $CD3^+$ $CD8^+$ population **(b)**, frequency of Naïve ($CD44^-$ $CD62L^+$), Effector ($CD44^-$ $CD62L^-$), Effector Memory ($CD44^+$ $CD62L^-$), and central memory (T_{CM}, $CD44^+$ $CD62L^+$) $CD8^+$ T cells **(c)**, and frequency of IFN-γ^+ **(d)** and TNF-α^+ **(e)** $CD8^+$ T cells.

Supplementary Figure 7

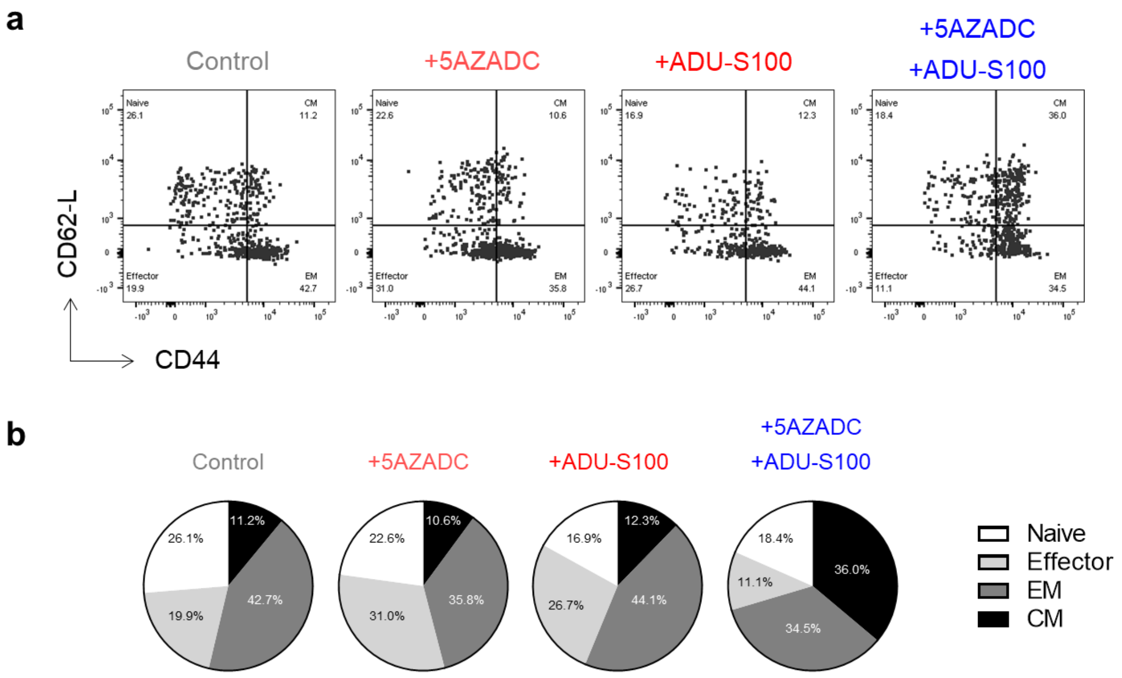

Supplementary Figure 7. CD8$^+$ TILs in combination therapy-treated mice indicate memory phenotype.

STING$^{gt/gt}$ mice with Yumm1.7 tumors were treated intratumorally with PBS, 5AZADC, ADU-S100, or 5AZADC+ADU-S100 as indicated in Figure 4a. Shown are representative flow cytometry plots **(a)** and pie charts **(b)** indicating relative proportions of Naïve (CD44$^-$ CD62L$^+$), Effector (CD44$^-$ CD62L$^-$), Effector Memory (EM; CD44$^+$ CD62L$^-$) and Central Memory (CM; CD44$^+$ CD62L$^+$) T cell subsets within the CD3$^+$ CD8$^+$ population in Yumm1.7 tumors on day 21.

Supplementary Figure 8

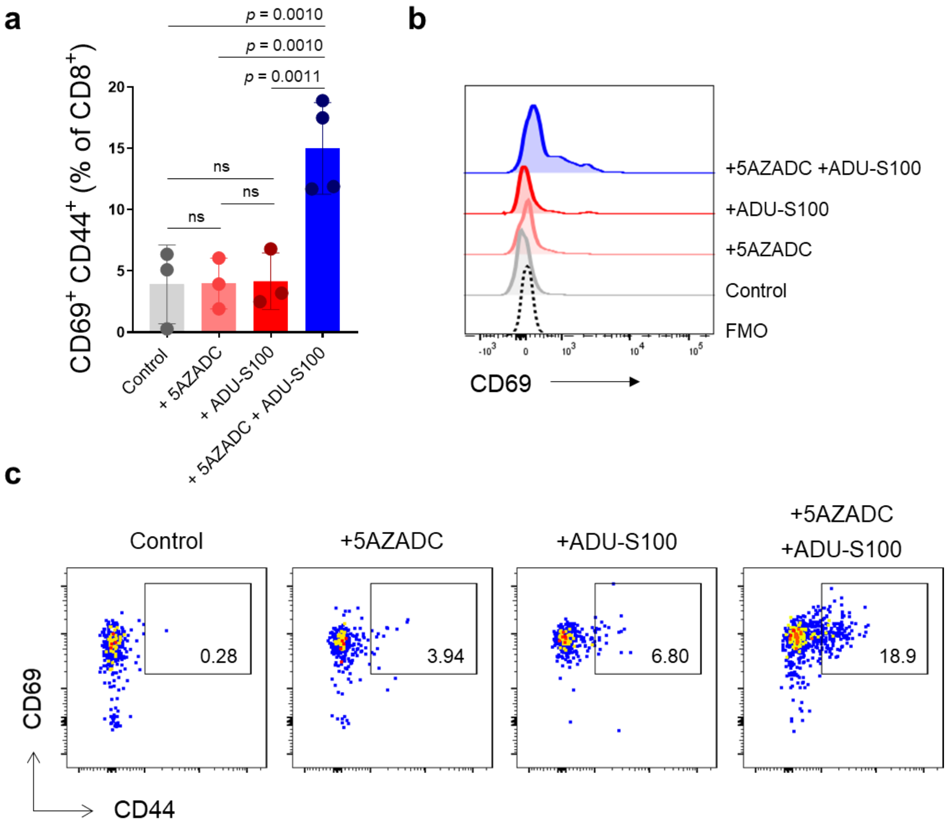

Supplementary Figure 8. CD8^{+} TILs in combination therapy-treated mice indicate higher expression of antigen-experience and activation markers.

STING$^{gt/gt}$ mice with B16-ISG tumors were treated intratumorally with PBS, 5AZADC, ADU-S100, or 5AZADC+ADU-S100 as indicated in Figure 4a. Frequency of CD44^{+} CD69^{+} cells **(a)** and representative histograms of CD69 **(b)** within the CD3^{+} CD8^{+} population in B16-ISG tumors on day 21. n = 3, 3, 3, 4 mice in (b) for Control, 5AZADC, ADU-S100, and 5AZADC+ADU-S100 groups, respectively. Data are shown as mean ± SD. Statistical significance was determined by one-way ANOVA (ns, not significant). Representative flow cytometry plots for (a) are shown in **(c)**.

Supplementary Figure 9

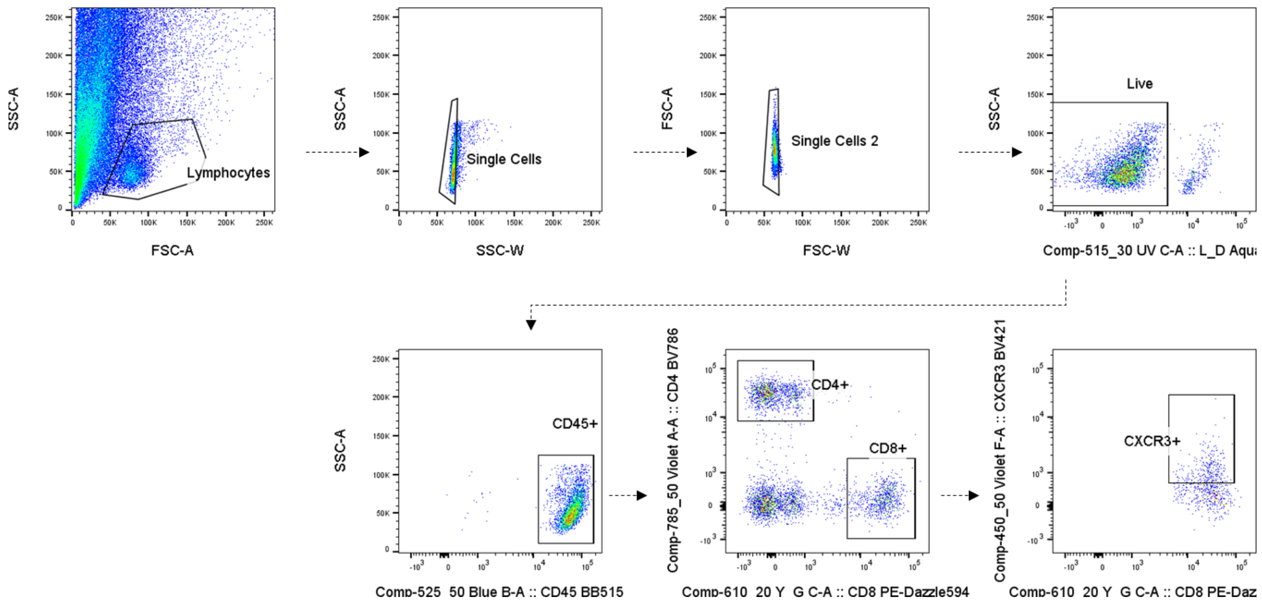

Supplementary Figure 9. Example of flow cytometry gating strategy.

Representative gating strategy to analyze $CD8^+$ T cells in tumors and spleens (relevant to Fig. 1d-g, Fig. 5b, Supplementary Fig. 1c-d, and Supplementary Fig. 5).

Supplementary Figure 10

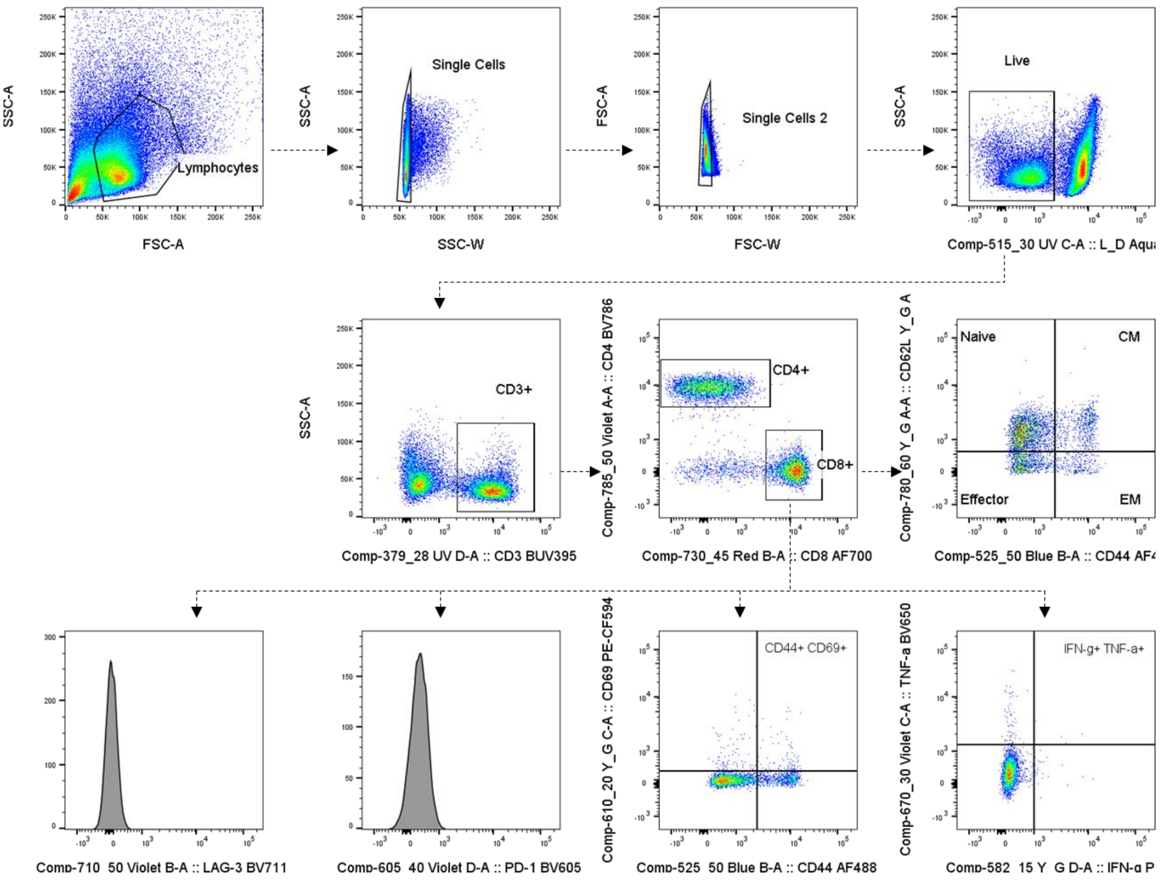

Supplementary Figure 10. Example of flow cytometry gating strategy.

Representative gating strategy to determine frequency, differentiation [Naïve: $CD44^-$ $CD62L^+$; Effector: $CD44^-$ $CD62L^-$; Effector Memory (EM): $CD44^+$ $CD62L^-$; Central Memory (CM): $CD44^+$ $CD62L^+$], activation ($CD44^+$ $CD69^+$) and IFN-γ and TNF-α expression of $CD8^+$ T cells in spleens and tumors (relevant to Fig. 6a-h, Fig. 7a-g, Fig. 8d-f, Supplementary Fig. 6a-e, Supplementary Fig. 7a-b, and Supplementary Fig. 8a-c)
