# Supplementary material for: Epigenetic state determines the in vivo efficacy of STING agonist therapy
Source: Nat Commun. 2023 Mar 22;14:1573. doi: 10.1038/s41467-023-37217-1 (PMC10033671; doi:10.1038/s41467-023-37217-1)
Supplement: Supplementary file 2 — Reporting Summary [file 41467_2023_37217_MOESM2_ESM.pdf]

nature portfolio

Corresponding author(s): James Mulé

Last updated by author(s): Feb 17, 2023

Reporting Summary

Nature Portfolio wishes to improve the reproducibility of the work that we publish. This form provides structure for consistency and transparency in reporting. For further information on Nature Portfolio policies, see our Editorial Policies and the Editorial Policy Checklist.

Statistics

For all statistical analyses, confirm that the following items are present in the figure legend, table legend, main text, or Methods section.

n/a	Confirmed	
☐	☒	The exact sample size (*n*) for each experimental group/condition, given as a discrete number and unit of measurement
☐	☒	A statement on whether measurements were taken from distinct samples or whether the same sample was measured repeatedly
☐	☒	The statistical test(s) used AND whether they are one- or two-sided<br>*Only common tests should be described solely by name; describe more complex techniques in the Methods section.*
☐	☒	A description of all covariates tested
☐	☒	A description of any assumptions or corrections, such as tests of normality and adjustment for multiple comparisons
☐	☒	A full description of the statistical parameters including central tendency (e.g. means) or other basic estimates (e.g. regression coefficient) AND variation (e.g. standard deviation) or associated estimates of uncertainty (e.g. confidence intervals)
☐	☒	For null hypothesis testing, the test statistic (e.g. *F*, *t*, *r*) with confidence intervals, effect sizes, degrees of freedom and *P* value noted<br>*Give P values as exact values whenever suitable.*
☒	☐	For Bayesian analysis, information on the choice of priors and Markov chain Monte Carlo settings
☒	☐	For hierarchical and complex designs, identification of the appropriate level for tests and full reporting of outcomes
☐	☒	Estimates of effect sizes (e.g. Cohen's *d*, Pearson's *r*), indicating how they were calculated

Our web collection on statistics for biologists contains articles on many of the points above.

Software and code

Policy information about availability of computer code

Data collection	BD FACSDiva software (BD Biosciences); GenomeStudio (V2011.1 Illumina Inc.)
Data analysis	FlowJo v10.7.1; GraphPad Prism v9 (GraphPad Software Inc.); ImageJ software (NIH); MATLAB (R2020a, Natick)

For manuscripts utilizing custom algorithms or software that are central to the research but not yet described in published literature, software must be made available to editors and reviewers. We strongly encourage code deposition in a community repository (e.g. GitHub). See the Nature Portfolio guidelines for submitting code & software for further information.

Data

Policy information about availability of data

All manuscripts must include a data availability statement. This statement should provide the following information, where applicable:

- Accession codes, unique identifiers, or web links for publicly available datasets
- A description of any restrictions on data availability
- For clinical datasets or third party data, please ensure that the statement adheres to our policy

All study data are included in the article and/or supplementary information files. Genomic data are from the cBioPortal repository (https://www.cbioportal.org/study/summary?id=skcm_mskcc_2014). Source data are provided with this paper.

Human research participants

Policy information about studies involving human research participants and Sex and Gender in Research.

Reporting on sex and gender	not applicable
Population characteristics	not applicable
Recruitment	not applicable
Ethics oversight	not applicable

Note that full information on the approval of the study protocol must also be provided in the manuscript.

Field-specific reporting

Please select the one below that is the best fit for your research. If you are not sure, read the appropriate sections before making your selection.

[x] Life sciences [ ] Behavioural & social sciences [ ] Ecological, evolutionary & environmental sciences

For a reference copy of the document with all sections, see nature.com/documents/nr-reporting-summary-flat.pdf

Life sciences study design

All studies must disclose on these points even when the disclosure is negative.

Sample size	All sample sizes are similar to those reported in previous publications (PMID: 32198222 and 34800368). All experiments were performed in multiple distinct replicates as indicated in the methods and figure legends.
Data exclusions	No data was excluded, except when samples did not pass the QC (i.e. cell viability/ cell recovery).
Replication	Experimental data reproduced in at least two independent experiments
Randomization	For in vivo experiments, mice were allocated randomly into two or four groups before receiving different treatments.
Blinding	Methylation microarray experiment (Fig. 2b-c) was performed blinded, until the final analysis by the biostatisticians. Most of the in vitro and in vivo experiments involved periodic treatment with different agents (e.g. 5AZADC, ADU-S100) at different time points. For these studies, we performed non-blinded treatments.

Reporting for specific materials, systems and methods

We require information from authors about some types of materials, experimental systems and methods used in many studies. Here, indicate whether each material, system or method listed is relevant to your study. If you are not sure if a list item applies to your research, read the appropriate section before selecting a response.

Materials & experimental systems

n/a	Involved in the study	
[ ]	[x]	Antibodies
[ ]	[x]	Eukaryotic cell lines
[x]	[ ]	Palaeontology and archaeology
[ ]	[x]	Animals and other organisms
[x]	[ ]	Clinical data
[x]	[ ]	Dual use research of concern

Methods

n/a	Involved in the study	
[x]	[ ]	ChIP-seq
[ ]	[x]	Flow cytometry
[x]	[ ]	MRI-based neuroimaging

Antibodies

Antibodies used	Anti-mouse antibodies used for Flow Cytometry (clone, dilution, supplier, cat#):<br>anti-CD45 BB515 (30-F11, 1:200, BD Biosciences, 564590)<br>anti-CD3e BUV395 (145-2C11, 1:50, BD Biosciences, 565533)<br>anti-CD4 BV786 (GK1.5, 1:100, BD Biosciences, 563331)<br>anti-CD8a PE/Dazzle 594 (53-6.7, 1:200, Biolegend, 100762)

	anti-CD8a Alexa Fluor 700 (53-6.7, 1:200, Biolegend, 100730)<br>anti-CD183 BV421 (CXCR3-173, 1:200, BD Biosciences, 566283)<br>anti-CD62L PE/Cy7 (MEL-14, 1:100, Biolegend, 104418)<br>anti-CD44 Alexa Fluor 488 (IM7, 1:200, Biolegend, 103016)<br>anti-CD69 PE-CF594 (H1.2F3, 1:100, BD Biosciences, 562455)<br>anti-LAG3 BV421 (C9B7W, 1:200, Biolegend, 125221)<br>anti-CD279 (PD-1) BV605 (J43, 1:50, BD Biosciences, 563059)<br>anti-TNF BV650 (MP6-XT22, 1:10, BD Biosciences, 563943)<br>anti-IFN-γ PE (XMG1.2, 1:10, BD Biosciences, 554412)<br>anti-H-2Kb Alexa Fluor 647 (AF6-88.5, 1:100, Biolegend, 116512)<br><br>anti-CD16/CD32 (Fc Block) (dilution: 1:50, Supplier: BD Bioscience, cat#: 553142)<br><br>Antibodies used for in vivo depletion studies (clone, amount, supplier, cat#):<br>InVivoMAb anti-mouse CD8α (2.43, 300 μg, Bio X Cell, BE0061)<br>InVivoMAb anti-mouse CD4 (GK1.5, 300 μg, Bio X Cell, BE0003-1)<br><br>Antibodies used for Western Blot (clone, dilution, supplier, cat#):<br>STING Rabbit mAb (D2P2F, 1:1000, Cell Signaling, 13647S)<br>DNMT1 (D63A6) XP® Rabbit mAb (D63A6, 1:1000, Cell Signaling, 5032S)<br>DNMT3A Rabbit mAb (D23G1, 1:1000, Cell Signaling, 3598S)<br>DNMT3B Rabbit mAb (D7O7O, 1:1000, Cell Signaling, 67259S)<br>LMP2 Rabbit mAb (EPR22042, 1:1000, Abcam, ab3328)<br>α-Tubulin Mouse mAb (DM1A, 1:5000, Cell Signaling, 3873S)<br>β-Actin Mouse mAb (AC-74, 1:5000, Sigma-Aldrich, A5316)<br><br>Secondary antibodies used for Western Blot (dilution, Supplier, cat#):<br>anti-rabbit IgG (1:2000, Cell Signaling, 7074S)<br>anti-mouse IgG (1:2000, Cell Signaling, 7076S)
Validation	The antibodies employed in our study were validated by the manufacturers and used according to the manufacturers' instructions. Flow cytometry antibodies were re-validated by titrating their concentrations and evaluating the staining efficiency in comparison with negative controls.

Eukaryotic cell lines

Policy information about cell lines and Sex and Gender in Research

Cell line source(s)	B16-F10 (mouse melanoma cell line) was purchased from American Type Culture Collection (ATCC). B16-ISG and B16-ISG-STINGKO (mouse melanoma cell lines) were purchased from InvivoGen. A375 and SK-MEL-28 (human melanoma cell lines, original source: ATTC) and Yumm1.7 (mouse melanoma cell line, original source: ATTC) were obtained from Dr. Keiran Smalley (Moffitt Cancer Center).
Authentication	Cell lines were authenticated by the companies we obtained them from. We checked the morphology of cell lines in culture and used low-passage cell cultures for all the experiments.
Mycoplasma contamination	Cell lines were routinely screened to avoid mycoplasma contamination. All cell lines tested negative for mycoplasma contamination.
Commonly misidentified lines<br>(See ICLAC register)	No commonly misidentified cell line was used in the study.

Animals and other research organisms

Policy information about studies involving animals; ARRIVE guidelines recommended for reporting animal research, and Sex and Gender in Research

Laboratory animals	Wild-type C57BL/6 and STINGgt/gt (Goldenticket; C57BL/6J-Sting1gt/J) mice were obtained from The Jackson Laboratory. All experiments were initiated using female mice between the ages of 8 and 10 weeks. Animals were housed in the Comparative Medicine Facilities at the Moffitt Cancer Center under temperature and humidity-controlled conditions with a 12-h light/dark cycle.
Wild animals	The study did not involve wild animals.
Reporting on sex	Sex was not considered in the study design. Female mice were used in all experiments.
Field-collected samples	The study did not involve samples collected from the field.
Ethics oversight	All animal work was approved by the Institutional Animal Care and Use Committee at the University of South Florida and performed in accordance with the U.S. Public Health Service policy and National Research Council guidelines.

Note that full information on the approval of the study protocol must also be provided in the manuscript.

Flow Cytometry

Plots

Confirm that:

- [x] The axis labels state the marker and fluorochrome used (e.g. CD4-FITC).
- [x] The axis scales are clearly visible. Include numbers along axes only for bottom left plot of group (a 'group' is an analysis of identical markers).
- [x] All plots are contour plots with outliers or pseudocolor plots.
- [x] A numerical value for number of cells or percentage (with statistics) is provided.

Methodology

Sample preparation	Single-cell suspensions of spleens were generated by passing cells through a 40-µm cell strainer. Red blood cells (RBCs) were removed from spleens using RBC lysis buffer (BioLegend). Tumor cell suspensions were prepared by enzymatic digestion in Hanks' Balanced Salt Solution (HBSS; Life Technologies) containing 1 mg/ml collagenase IV, 0.1 mg/ml DNaseI, and 2.5 U/ml hyaluronidase (all from Sigma-Aldrich) and then subjected to GentleMACS dissociation (Miltenyi Biotec). Tumor digest cell suspensions were incubated at 37°C in a rocking water bath for 1 h. RBCs were removed using RBC Lysis Buffer (BioLegend), then cell suspensions were filtered with a 100-µm cell strainer to remove large cellular debris.
Instrument	Samples were collected on an LSRII cytometer (BD Biosciences).
Software	Flow cytometry data were collected using BD FACSDiva version 9 software (BD Biosciences) and analyzed with FlowJo v10.7.1 software (Tree Star Inc.).
Cell population abundance	We did not perform any sorting. Not applicable.
Gating strategy	Gating strategy is described in the figure legend of each figure reporting flow cytometry data. Gating schemes are provided in the Supplementary Information.

- [x] Tick this box to confirm that a figure exemplifying the gating strategy is provided in the Supplementary Information.
